# Reduction in myofilament Ca^2+^ sensitivity partially ameliorates the cardiac phenotype in hypertrophic cardiomyopathy linked to a TnT-R92Q mutation

**DOI:** 10.3389/fphys.2025.1600117

**Published:** 2025-05-23

**Authors:** Paulina Langa, Angelie Bacon, Chad M. Warren, Shamim A. K. Chowdhury, Monika Halas, Aurelia A. Fernandes, Mark D. McCauley, Paul H. Goldspink, R. John Solaro, Beata M. Wolska

**Affiliations:** ^1^ Department of Physiology and Biophysics, College of Medicine, University of Illinois, Chicago, IL, United States; ^2^ Center for Cardiovascular Research, College of Medicine, University of Illinois, Chicago, IL, United States; ^3^ Department of Medicine, Division of Cardiology, College of Medicine, University of Illinois, Chicago, IL, United States; ^4^ Department of Medicine, Jesse Brown VA Medical Center, Chicago, IL, United States

**Keywords:** hypertrophic cardiomyopathy, myofilament Ca^2+^ sensitivity, fibrosis, YAP signaling, coronary flow, thin filaments

## Abstract

Hypertrophic cardiomyopathy (HCM) is a genetic disease caused by mutations in sarcomeric proteins. Mutations in sarcomeric proteins that give rise to cardiomyopathies produce abnormalities in the biophysical properties of the sarcomere that are propagated beyond the cardiac myocyte. In this study, we evaluated the hypothesis that early desensitization of myofilament Ca^2+^ sensitivity in the TnT-R92Q mouse model, achieved through the introduction of pseudo-phosphorylated TnI (TnI-S23,24D or TnI-DD), may delay the progression of the HCM phenotype in cardiac myocyte and endothelial cellular compartments of the heart. We studied non-transgenic mice, transgenic (TG) mice expressing TnT-R92Q (TnT-R92Q), TG mice expressing TnI-DD, and double transgenic mice expressing TnT-R92Q and TnI-DD at 28 days and 16 weeks of age. Experiments reported here demonstrate that expression of TnI-DD in the TnT-R92Q HCM mouse model results in partial normalization of myofilament Ca^2+^ sensitivity, improved cardiac morphology and function, reduced fibrosis, normalization of YAP signaling in endothelial cells, but a lack of normalization of coronary flow parameters. The novelty of the approach reported here highlights that although small corrections made to offset the sarcomeric defect may not fully or immediately resolve the disease’s pathophysiologic state, they can lessen the severity of HCM. Our findings further support the concept that early desensitization of myofilaments to Ca^2+^ in HCM, mainly when arising from mutations in thin filament proteins, represents a promising avenue for developing new therapeutic drugs.

## 1 Introduction

Hypertrophic cardiomyopathy (HCM) is recognized as the most prevalent inherited cardiac condition, occurring in approximately 1:200–1:500 individuals within the general population (Semsarian et al., 2015). It stands as the primary inherited cause of atrial fibrillation, ventricular arrhythmias, and sudden cardiac death (SCD), particularly among young competitive athletes (Maron, 2009; Maron, 2010; D’Ascenzi et al., 2022). Most HCM cases, about 60%–70%, are attributed to mutations in sarcomeric or cytoskeletal proteins. The distinctive features of HCM include unexplained left ventricular (LV) hypertrophy, often asymmetrical, cardiomyocyte disarray, and patchy fibrosis. Approximately 70% of sarcomeric mutations are associated with genes that encode thick filament proteins, specifically myosin-binding protein C (MYBPC3), beta myosin heavy chain 7 (MYH7), and light chains (*MYL2* and *MYL3*). A notable proportion of mutations, ranging from 1% to 5%, have been detected in genes responsible for thin filament proteins: troponin T (*TNNT2*), troponin I (*TNNI3*), tropomyosin (*TPM1*), and actin (*ACTC1*) (Akhtar and Elliott, 2018). Mutations in other sarcomeric-associated proteins account for less than 1% of all HCM-identified variants (Ommen et al., 2020).

We have previously reported that interventions aimed at enhancing myofilament relaxation in the early stages of HCM in mouse models with thin filament mutations in tropomyosin (Tm-E180G) and troponin T (TnT-R92Q) delay disease progression and improve cardiac function (Pena et al., 2010; Gaffin et al., 2011; Alves et al., 2014; Chowdhury et al., 2020). Our recent findings revealed that during the initial phase of HCM development in TnT-R92Q models, increased myofilament Ca^2+^ sensitivity leads to diastolic dysfunction, transient changes in coronary flow, and premature fibrosis (Langa et al., 2023). The study of TnT mutations is critical; adults with HCM-related TnT mutations exhibit a more severe prognosis despite less pronounced hypertrophy than those with thick filament mutations (Coppini et al., 2014). Furthermore, the likelihood of heart failure-associated conditions is greater in cases involving thin filament mutations than in those linked to thick filament HCM mutations (van Velzen et al., 2016).

Specific characteristics of the outcomes in patients with HCM-linked mutations in genes that express thin filament proteins suggest that further research is necessary to comprehend the progression of this pathology, particularly in its initial stages. Up to 20% of children with HCM-linked variants of sarcomere protein mutations harbor gene variants encoding thin filament proteins (Norrish et al., 2024). Thus, there is a need for a deeper understanding of triggers for adverse events in younger patients and potential therapies. A clue to the primary trigger is that a property of the HCM-sarcomeres is frequently an increase in myofilament Ca^2+^ sensitivity (Willott et al., 2010), commonly linked to altered diastolic function. Recently, Spudich et al. suggested that nearly all myosin HCM mutations show an increased number of ON-state myosin compared to controls (Spudich et al., 2024). Importantly, most thin filament protein mutations alone are sufficient to enhance myofilament Ca^2+^ sensitivity. Notably, in HCM, signs of diastolic dysfunction may be present even without hypertrophy or other clinical pathologies (Poutanen et al., 2006; Ho et al., 2009). Such findings have been substantiated through studies with human tissue samples, animal models, and *in vitro* experiments with reconstituted myofilament preparations (Muthuchamy et al., 1999; Szczesna et al., 2000; Chandra et al., 2001; Sequeira et al., 2013). Currently, mavacamten (marketed as Camzyos®) is the sole medication sanctioned for treating adult symptomatic obstructive HCM (Keam, 2022). However, this small-molecule inhibitor of myosin is exclusively authorized for use in adults with obstructive HCM (Braunwald et al., 2023). In recent developments, another myosin inhibitor, Aficamten, has shown promising outcomes in the REDWOOD-HCM (Owens et al., 2023) and SEQUOIA-HCM trials (Coats et al., 2024). Given that both drugs are myosin inhibitors targeting the effects of mutations in thick filament proteins, developing new treatments for HCM patients, especially the pediatric population, focusing on thin filament mutations, remains imperative. Directly targeting the Ca^2+^ sensitivity of myofilaments could represent a viable therapeutic avenue.

In this study, we evaluated the hypothesis that early desensitization of myofilament Ca^2+^ sensitivity in the TnT-R92Q mouse model, achieved through the introduction of pseudo-phosphorylated TnI (TnI-DD), may delay the onset of the HCM phenotype by the normalization of diastolic function, atrial size, reduction of fibrosis, and improved coronary flow dynamics. Our findings further support the concept that early desensitization of myofilaments to Ca^2+^ in HCM, mainly when arising from mutations in thin filament proteins, represents a promising avenue for developing new therapeutic drugs.

## 2 Methods

Expanded materials and methods can be found in the Supplementary Material.

### 2.1 Institutional approval and animal model

New transgenic (TG) mouse lines were generated by crossbreeding existing lines of mice, TnT-R92Q (Tardiff et al., 1999; Chowdhury et al., 2020) and TnI-S23,24D (TnI-DD) (Sakthivel et al., 2005). All mice used in this work were in the FVB/N genetic background (Charles River). Four groups of mice were used for experiments: (1) non-transgenic (NTG) mice, which express wildtype troponin T and wildtype troponin I; (2) TnT-R92Q; (3) TnI-DD; (4) TnT-R92Q/TnI-DD (double transgenic; DTG), which express both transgenic variants, TnT-R92Q and TnI-DD (Supplementary Figure S1). Experiments were performed with 28-day and 4-month-old animals. This study was approved by the Institutional Animal Care and Use Committee (IACUC) of the University of Illinois at Chicago, accredited by the American Association for the Accreditation of Laboratory Animal Care (AAALAC). All animals received humane care in compliance with the “Principles of Laboratory Animal Care” formulated by the National Society for Medical Research and the “Guide for the Care and Use of Laboratory Animal Resources.” Animals were kept in pathogen-free environments on a light/dark cycle.

### 2.2 Skinned fiber bundles tension measurement

Hearts were excised, and detergent-extracted (skinned) fiber bundles were prepared for force-Ca^2+^ measurements as described previously (Alves et al., 2014).

### 2.3 SDS-PAGE and immunoblotting

Whole-heart homogenates and isolated myofibrils from frozen hearts were prepared with a Bead Ruptor 24 Elite, as outlined previously (Batra et al., 2021; Capote et al., 2021). To separate isoforms of myosin heavy chain (MHC), a 6% SDS-PAGE was utilized (Warren and Greaser, 2003), and for determining phosphorylation abundances of regulatory light chain (RLC), PhosTag gels (Kinoshita et al., 2006) were run as described in Supplementary Material. All other SDS-PAGE gels for Western blotting or Pro-Q Diamond staining were 12% or 15%, as described in the Supplementary Material. The abundance levels were analyzed with Image Lab v6.0.1 (BioRad).

### 2.4 Echocardiography

Transthoracic echocardiography was performed using a Vevo 2100 High-Resolution *In Vivo* Imaging System (FUJIFILM VisualSonics, 2100) as previously described (Alves et al., 2014; Chowdhury et al., 2020). In addition, left coronary flow measurements were performed after morphometric and functional cardiac assessment as described by Chang et al. (2015). All measurements and calculations were averaged from three consecutive cycles and performed according to the American Society of Echocardiography guidelines. Data analysis was performed with the VevoLab ver. 5.5.1. Analytic Software.

### 2.5 Histology and immunohistochemistry

The heart samples were fixed in 10% formalin for fibrosis assessment, followed by paraffin embedding, and sectioned as previously described (Alves et al., 2014). Formalin-fixed and paraffin-embedded (FFPE) sections were de-paraffinized, rehydrated, and antigen-retrieved (Tris-EDTA), followed by blocking (10% BSA). To assess fibrosis, sections were stained with a Picro Sirius Staining kit for cardiac muscle (Abcam, cat. ab245887) to visualize collagenous fibrotic tissue. Pixels corresponding to the area stained red, indicating collagenous areas reflecting fibrosis, were normalized to the total pixel area of the tissue in the assessed image (yellow). Images of whole heart sections and regions of interest were taken using a Zeiss Axio Imager Z2 (Germany) brightfield microscope.

Sections were incubated in anti-CD31, anti-αSMA, and anti-YAP antibodies for immunohistochemistry. After washing with PBS, they were incubated with secondary antibodies, followed by DAPI for nuclear counterstaining. Images were acquired with the Zeiss LSM880 confocal microscope (Germany) equipped with a motorized stage (see the Supplementary Material for details).

### 2.6 Statistical analysis

All statistical analysis was performed using GraphPad Prism v. 9.3.1 or 10.0.3. Data are presented as mean ± SEM (standard error of the mean), n = number of samples. Gaussian distribution was assessed by the Kolmogorov-Smirnov test unless otherwise stated in the figure or table legends, and the homogeneity of variance was assessed by the Brown-Forsythe test. One-way ANOVA followed by Tukey’s multiple comparison test was used for normally distributed and equal SD groups. The Dunnett T3 test was used when the groups showed a normal distribution and no equal SDs. The Kruskal–Wallis test was used, followed by the Kruskal–Wallis test when the data failed normal distribution. Unpaired two-tailed Student’s *t*-tests were performed when two groups were compared. All statistical analysis was performed using GraphPad Prism v. 9.3.1 or 10.0.3. Detailed information on the statistical tests performed is presented in the Figure and Table legends. Significance was set to P < 0.05.

## 3 Results

### 3.1 Expression of TnI-DD in TnT-R92Q hearts prevents pathological remodeling and improves LV diastolic function

**FIGURE 1:**
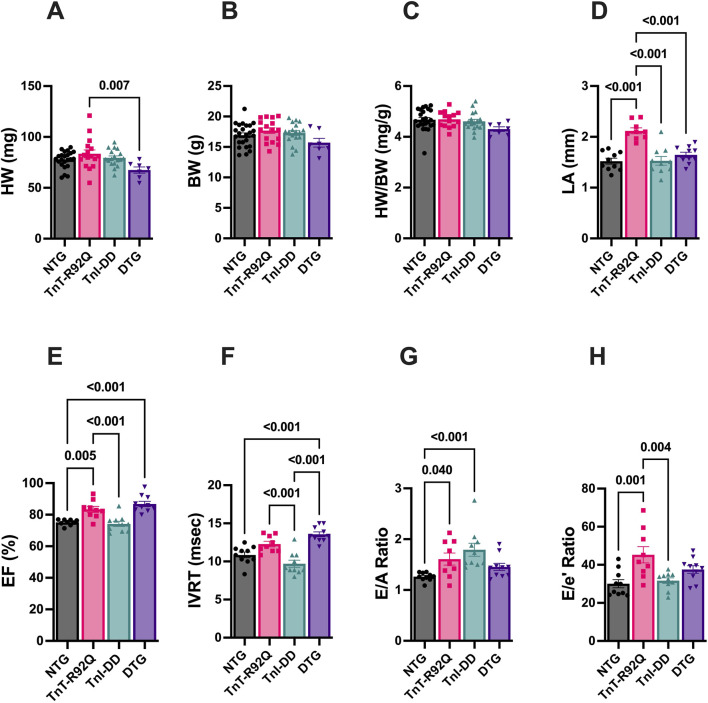
Morphological, systolic, and diastolic parameters in NTG, TnT-R92Q, TnI-DD, and DTG hearts at 28 days of age. **(A)** Heart weight (HW), **(B)** body weight (BW), **(C)** heart weight to body weight (HW/BW), **(D)** left atrial diameter (LA), **(E)** ejection fraction (EF), **(F)** isovolumic relaxation time (IVRT), **(G)** E/A ratio represents peak velocity of early diastolic mitral flow divided by peak velocity of late diastolic mitral inflow, **(H)** E/e’ ratio represents peak velocity of early diastolic transmitral flow divided by peak velocity of early diastolic mitral annual motion. Data are reported as mean ± SEM. *n* = 9–10 per group; Data were analyzed by 1-way ANOVA followed by the Tukey’s multiple comparisons test **(A–F,H)**; E/A ratio data **(G)** were analyzed by the Kruskal–Wallis ANOVA test followed by the Dunn’s multiple comparisons test. NTG, non-transgenic; TnT-R92Q, transgenic mice expressing TnT-R92Q; TnI-DD, transgenic mice expressing TnI-S23,24D; DTG, double transgenic.

We have previously reported that TnT-R92Q mice demonstrate cardiac remodeling and diastolic dysfunction as early as 7–14 days of age, and both remodeling and diastolic dysfunction progress with age (Langa et al., 2023). In this paper, we tested whether the expression of TnI-DD, which mimics the phosphorylation of S23 and S24 in the N-terminus of cTnI, can prevent or delay these early pathological changes. Figure 1A shows that at 28 days of age, there was no difference in heart weight (HW) between NTG and other groups. However, DTG hearts were slightly smaller than TnT-R92Q hearts. Left ventricular internal diastolic dimension (LVIDd), relative wall thickness (RWT), and left ventricular (LV) mass calculated from echocardiography were not different between groups (Supplementary Figures S2A–C). There were no differences in body weight (BW) (Figure 1B) or HW/BW (Figure 1C) between all four experimental groups. Left atrial (LA) size, assessed by echocardiography, was increased in TnT-R92Q mice but was normalized in DTG hearts (Figure 1D). TnT-R92Q hearts show a slight increase in ejection fraction (EF) compared to NTG hearts, which also persisted in DTG hearts (Figure 1E). This increase in EF in TnT-R92Q hearts was not associated with an increase in CO (Supplementary Figure S2D). However, CO was slightly reduced in the DTG group compared to TnI-DD and NTG mice. We observed that TnT-R92Q and DTG hearts had decreased HR compared to NTG (Supplementary Figure S2E). There were no changes in stroke volume (SV) between groups (Supplementary Figure S2F). TnT-R92Q mice showed an increased E/A and E/e’ ratios with no change in IVRT compared to NTG, which suggests the development of diastolic dysfunction (Figures 1F–H). However, DTG mice compared to NTG only show increased IVRT and no significant changes in E/A and E/e’ ratios, suggesting improved diastolic function.

**FIGURE 2:**
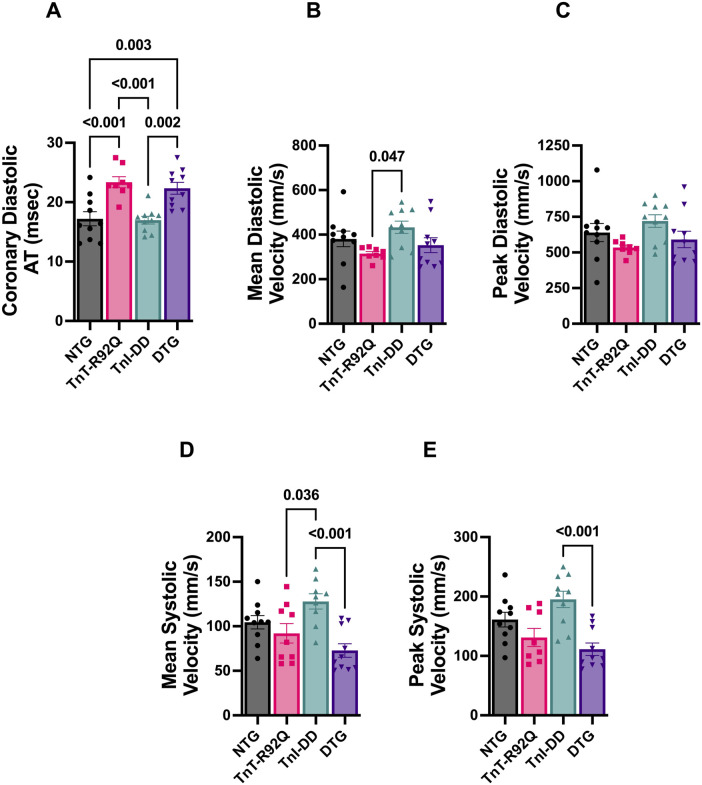
Coronary flow parameters in NTG, TnT-R92Q, TnI-DD, and DTG hearts at 28 days of age. **(A)** Diastolic acceleration time (AT), **(B)** mean diastolic velocity, **(C)** peak diastolic velocity, **(D)** mean systolic velocity, **(E)** peak systolic velocity. *n* = 8–10. Data were analyzed by 1-way ANOVA followed by Tukey’s multiple comparisons test **(A–D)**; peak systolic velocity data **(E)** were analyzed by the Kruskal–Wallis ANOVA test followed by the Dunn’s multiple comparisons test. NTG, non-transgenic; TnT-R92Q, transgenic mice expressing TnT-R92Q; TnI-DD, transgenic mice expressing TnI-S23,24D; DTG, double transgenic.

In addition to increased atrial size and diastolic dysfunction, TnT-R92Q mice show developmental changes in coronary flow dynamics during early development (Langa et al., 2023). Figure 2 indicates that most diastolic coronary flow parameters are similar between NTG and TnT-R92Q groups at 28 days of age, except for prolonged diastolic acceleration (rise) time (Figures 2A–C). Interestingly, this parameter was also prolonged in DTG mice compared to NTG and TnI-DD groups. There were no differences in systolic parameters between NTG and TnT-R92Q groups (Figures 2D,E). However, both mean and peak systolic velocities were significantly lower in DTG hearts compared to TnI-DD hearts. All the numerical echocardiography data and statistical test details are presented in Supplementary Tables S1,S2.

### 3.2 Expression of TnI-DD in TnT-R92Q mice reduces myofilament Ca^2+^ sensitivity and alters myofilament isoform expression and phosphorylation

**FIGURE 3:**
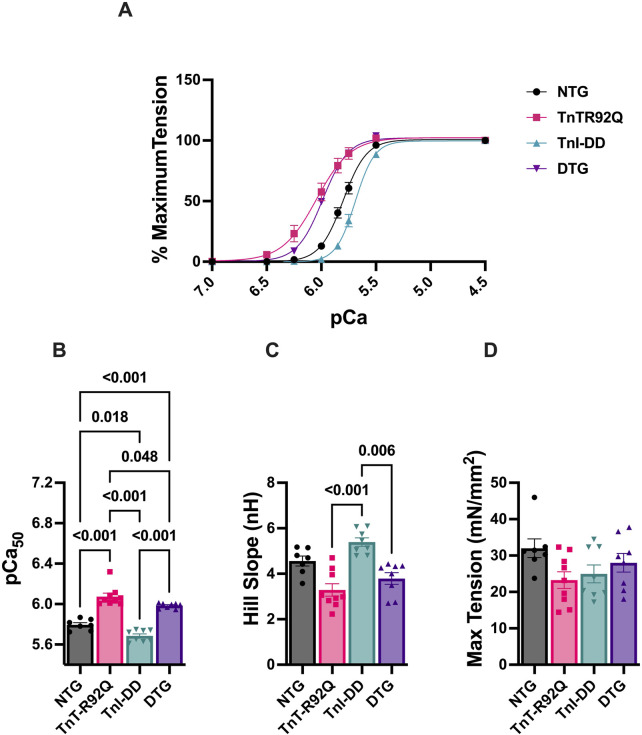
The myofilament Ca^2+^ response. **(A)** Normalized force-Ca^2+^ relation, **(B)** myofilament Ca^2+^ sensitivity (pCa_50_), **(C)** Hill slope, and **(D)** maximum tension. Data are presented as mean ± SEM. *n* = 8–9 pCa_50_ data **(A)** were analyzed by 1-way ANOVA followed by Tukey’s multiple comparisons test. The Hill slope and max tension data were analyzed using the Kruskal–Wallis ANOVA test, followed by Dunn’s multiple comparisons test. NTG, non-transgenic; TnT-R92Q, transgenic mice expressing TnT-R92Q; TnI-DD, transgenic mice expressing TnI-S23,24D; DTG, double transgenic.

Our previous studies in cTnT-R92Q mice showed increased myofilament Ca^2+^ sensitivity in the heart as early as 7 days of age (Langa et al., 2023). We also reported that crossing TnI-DD and Tm-E180G mice to express TnI-DD on the Tm-E180G background resulted in small desensitization of the myofilaments to Ca^2+^ (Alves et al., 2014). To determine the levels of myofilament Ca^2+^ sensitivity, we measured the force-Ca^2+^ relationship in skinned fiber bundles prepared from papillary muscles of NTG, TnT-R92Q, TnI-DD, and DTG mice (Figure 3A; Supplementary Table S3). Myofilament Ca^2+^ sensitivity (pCa_50_) was increased in TnT-R92Q (pCa_50_ = 6.07 ± 0.033, *n* = 9) and decreased in TnI-DD (pCa_50_ = 5.69 ± 0.020, *n* = 8) compared to the NTG (pCa_50_ = 5.73 ± 0.021, *n* = 7) group. Importantly, the Ca^2+^ sensitivity of fibers from DTG hearts (pCa_50_ = 5.98 ± 0.009, *n* = 8) was significantly lower than in TnT-R92Q mice (Figure 3B). The Hill coefficient of the pCa-force relationship was increased in the TnI-DD compared to the TnT-R92Q and DTG groups (Figure 3C), and maximum tension was decreased in the TnT-R92Q compared to the NTG group (Figure 3D). No significant changes in maximum tension were observed between the NTG and DTG groups.

**FIGURE 4:**
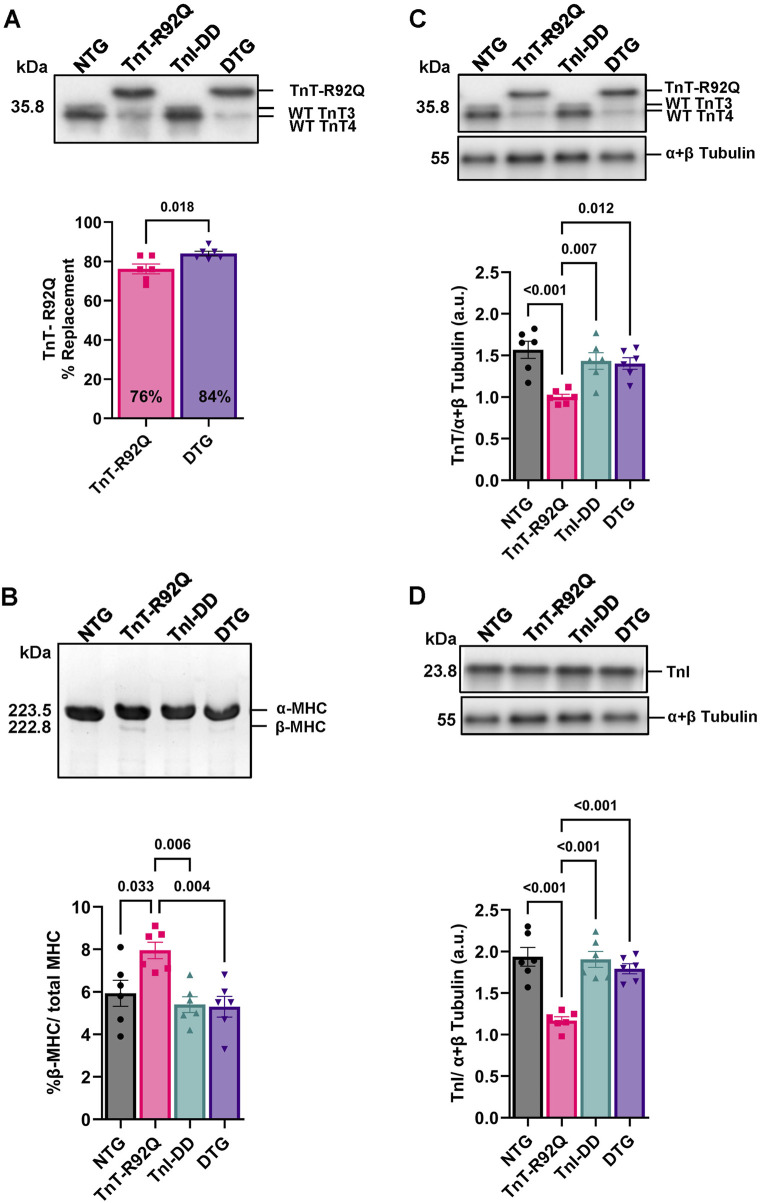
Replacement of total TnT by TnT-R92Q in TnT-R92Q and DTG whole heart homogenates, β-MHC, troponin T (TnT), and troponin I (TnI) abundance at 28 days of age. **(A)** A representative Western blot image of wildtype (WT) troponin T (TnT: TnT_3_ and TnT_4_) and the mutant form TnT-R92Q is shown with quantitation below. **(B)** Below is a representative SDS-PAGE image of myosin heavy chain (MHC) alpha and beta isoform separation and quantitation. **(C)** Representative Western blot images of TnT isolated myofilaments and α + β tubulin with quantitation below. **(D)** Representative Western blot images of TnI isolated myofilaments and α + β tubulin with quantitation below. Data are reported as mean ± SEM, *n* = 6. Data were analyzed by 1-way ANOVA followed by Tukey’s test (MHC) or an unpaired T-test (TnT-R92Q). NTG, non-transgenic; TnT-R92Q, transgenic mice expressing TnT-R92Q; TnI-DD, transgenic mice expressing TnI-S23,24D; DTG, double transgenic.

Since co-expression of TnI-DD with TnT-R92Q in the DTG mice may alter the expression of mutated TnT and contribute to the myofilament Ca^2+^ sensitivity, we assessed the levels of TnT-R92Q expression in both TnT-R92Q and DTG hearts (Figure 4A). We found a slight but significant increase in TnT-R92Q abundance in DTG hearts (84% ± 1.2%, *n* = 6) compared to TnT-R92Q hearts (76% ± 2.5%, *n* = 6). The re-expression of the β-MHC isoform is considered a hallmark of hypertrophic remodeling in rodent models for heart failure, and we have previously reported that the abundance of β-MHC protein was increased in TnT-R92Q mice (Chowdhury et al., 2020; Langa et al., 2023). Since the altered expression of MHC isoforms could contribute to the altered cardiac dynamics (Palmiter et al., 1999), we quantified the expression of MHC isoforms in all the groups (Figure 4B). Expression of α- and β-MHC isoforms was similar in NTG, TnI-DD, and DTG myofilaments. However, the expression of the β-MHC remained significantly elevated in myofilaments from TnT-R92Q hearts at 28 days compared to other groups.

Next, we compared the abundance of total TnT and TnI in isolated myofilament fractions. The total abundance of TnT was decreased in myofilaments from TnT-R92Q compared to NTG hearts (Figure 4C). There was no difference in TnT abundance in isolated myofilaments from the DTG compared to TnI-DD and NTG mice (Figure 4C). However, there was an increase in TnT abundance in DTG compared to TnT-R92Q hearts. We also found that TnI abundance was lower in myofilaments from TnT-R92Q hearts compared to other groups but normalized in DTG preparations (Figure 4D).

**FIGURE 5:**
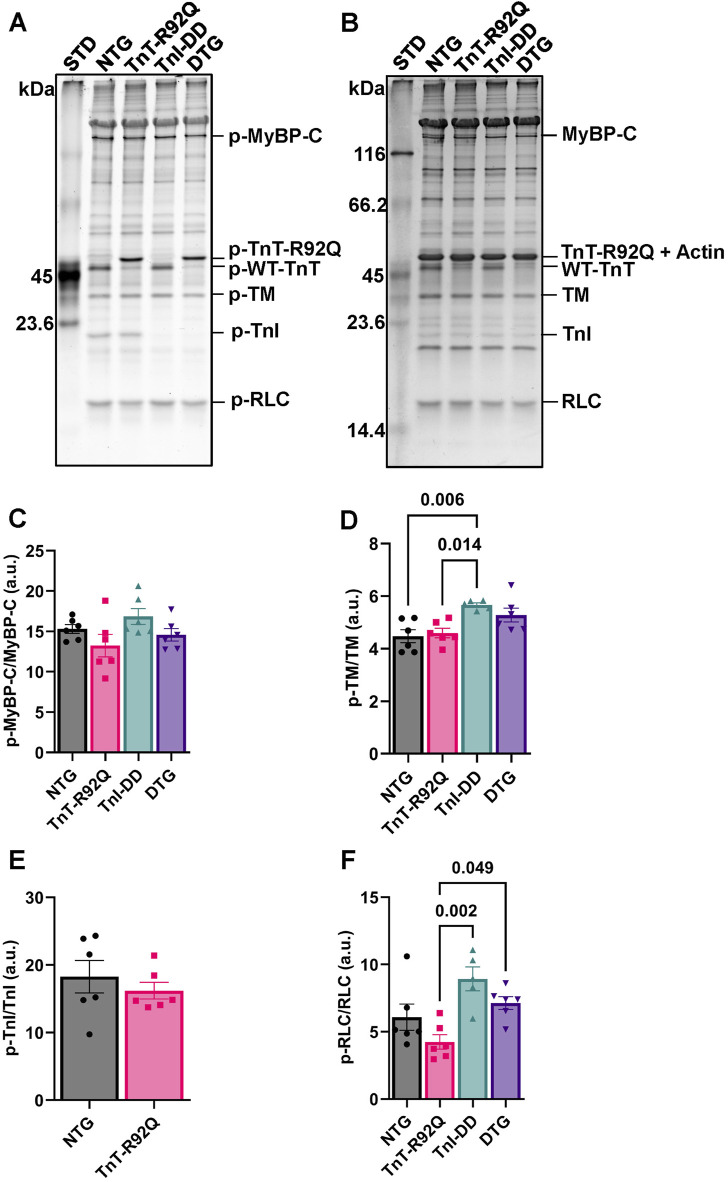
Phosphorylation (p) of myosin binding protein C (p-MyBPC), tropomyosin (TM), troponin I (TnI), and regulatory light chain (RLC) in isolated myofilaments via Pro-Q Diamond staining of SDS-PAGE. **(A)** Representative image of SDS-PAGE stained with Pro-Q Diamond phospho-specific stain. **(B)** A representative image of SDS-PAGE is stained with Coomassie stain. **(C)** Histogram of myosin binding protein C (MyBP-C) phosphorylation abundance. **(D)** Histogram of tropomyosin phosphorylation abundance. **(E)** Histogram of troponin I phosphorylation abundance. Note Pro-Q Diamond stain primarily stains the phosphorylated serine 22–23 of cardiac TnI, which was not detected in the TnI-DD and DTG groups. **(F)** Histogram of regulatory light chain phosphorylation abundance. Data are reported as mean ± SEM, *n* = 5–6. Data were analyzed by 1-way ANOVA followed by Tukey’s test or an unpaired *t*-test. NTG, non-transgenic; TnT-R92Q, transgenic mice expressing TnT-R92Q; TnI-DD, transgenic mice expressing TnI-S23,24D; DTG, double transgenic.

To account for changes in myofilament phosphorylation of other relevant sarcomere proteins, as a potential contributing underlying factor to the observed changes in the myofilament’s sensitivity to Ca^2+^, we performed Pro-Q Diamond staining of isolated myofilaments following SDS-PAGE (Figure 5). We detected no changes in total MyBP-C, TnI, TM, or RLC phosphorylation between NTG and TnT-R92Q groups (Figures 5C–F). An increase in TM phosphorylation was observed in the TnI-DD group compared to the NTG and TnT-R92Q groups, which was not evident in the DTG group (Figure 5D). In contrast, total RLC phosphorylation was decreased in the TnT-R92Q compared to TnI-DD and DTG groups (Figure 5F). To examine RLC phosphorylation further, we performed Western blot PhosTag separation (Supplementary Figure S3A) to quantify the different phospho-RLC sites (p1 – Supplementary Figure S3B, p2- Supplementary Figure S3C, and both sites–Supplementary Figure S3D). There was an increase in p1 and total phosphorylation of RLC in TnI-DD compared to NTG and TnT-R92Q mice, which were not apparent in the DTG mice.

### 3.3 Expression of TnI-DD in TnT-R92Q hearts alters the abundance and phosphorylation of Ca^2+^ regulatory proteins

**FIGURE 6:**
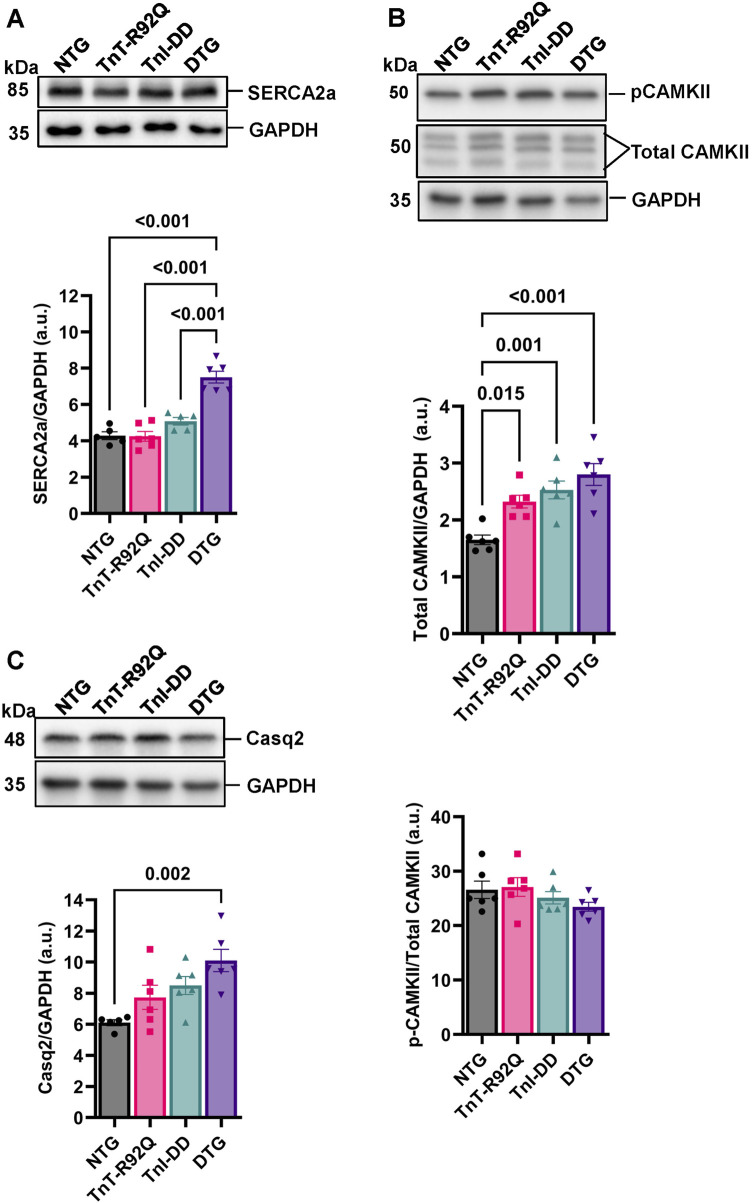
Ca^2+^ handling proteins abundance and phosphorylation. **(A)** Representative Western blot images of sarcoplasmic/endoplasmic reticulum Ca^2+^ ATPase 2a (SERCA2a) and GAPDH loading control with histogram below. **(B)** Representative Western blot images of calcium-calmodulin-dependent protein kinase II (CAMKII) and GAPDH loading control with histograms below. **(C)** Representative Western blot images of calsequestrin 2 (Casq2) and GAPDH loading control with histogram below. Data reported as mean ± SEM, *n* = 5–6. Data were analyzed by 1-way ANOVA followed by Tukey’s test. NTG, non-transgenic; TnT-R92Q, transgenic mice expressing TnT-R92Q; TnI-DD, transgenic mice expressing TnI-S23,24D; DTG, double transgenic.

We have previously reported that expressing TnI-DD in the Tm-E180G HCM mouse model results in increased phospholamban (PLN) phosphorylation without changes in either PLN or Serca2 abundance (Alves et al., 2014). Here, we found that DTG hearts showed no changes in total PLN expression compared to other groups (Supplementary Figures S4A–D) but did show increased p-PLN phosphorylation at Ser-16 (Supplementary Figures S4A,C,E) with no changes in Thr-17 phosphorylation (Supplementary Figures S3B,D,F). Interestingly, we also found an increased abundance of SERCA2a in the DTG group compared to other groups (Figure 6A). Total CAMKII abundance was increased in TnT-R92Q, TnI-DD, and DTG groups compared to the NTG group (Figure 6B), but with no change in CAMKII phosphorylation. In addition, the DTG hearts also showed a significant increase in Casq2 abundance compared to the NTG group (Figure 6C).

### 3.4 The expression of TnI-DD in TnTR92Q hearts results in the normalization of GATA4 phosphorylation but does not normalize the abundance of ERK1/2

**FIGURE 7:**
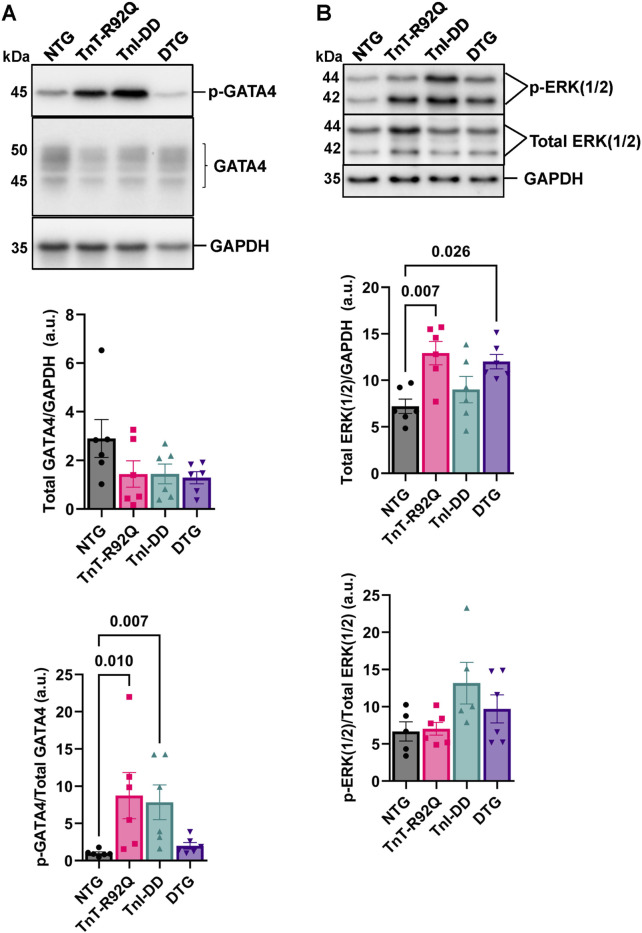
GATA4 (GATA Binding Protein 4) and extracellular signal-regulated kinase isoforms 1 and 2 (ERK1/2) expression and phosphorylation (p) abundance in whole heart homogenates. **(A)** Representative Western blot images of p-GATA4, GATA4, and GAPDH loading control with histograms below. **(B)** Representative Western blot images of the p-extracellular signal-regulated kinase isoforms 1 and 2 (ERK1/2), total ERK1/2, and GAPDH loading control with histograms below. Data are reported as mean ± SEM, *n* = 5–6. Data were analyzed by Kruskal–Wallis followed by Dunn’s test (GATA4) or 1-way ANOVA followed by Tukey’s test (ERK1/2). NTG, non-transgenic; TnT-R92Q, transgenic mice expressing TnT-R92Q; TnI-DD–transgenic mice expressing TnI-S23,24D; DTG, double transgenic.

Figure 7A shows no changes in GATA4 expression between the groups. However, we found increased phosphorylation of GATA4 in TnT-R92Q and TnI-DD hearts compared to NTG, which was unchanged in the DTG group. Interestingly, the expression of total ERK1/2 was increased in the TnT-R92Q group compared to NTG and was not normalized in the DTG group (Figure 7B). No significant changes in phosphorylation of ERK1/2 were observed between the groups.

### 3.5 YAP expression and localization changes in endothelial cells are normalized by TnI-DD expression in TnT-R92Q hearts

**FIGURE 8:**
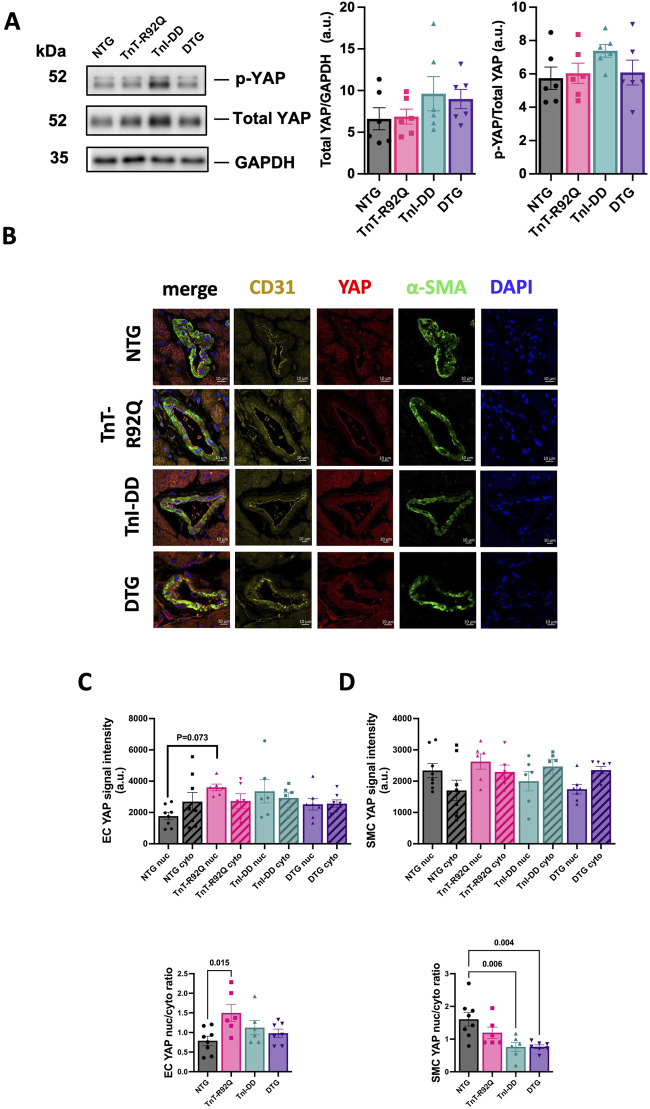
Expression and localization of YAP. **(A)** Representative Western blotting protein images for phospho-YAP and total YAP with a histogram to the right for total YAP/GAPDH and p-YAP/total YAP. **(B)** Representative images of heart sections showing YAP expression co-localized with αSMA, CD31, and DAPI counterstaining. **(C)** YAP signal assessment in EC cells (top histogram), and nuclear/cytoplasmic ratio of YAP signal in EC cells (lower histogram). **(D)** YAP signal assessment in smooth muscle cells (top histogram), and nuclear/cytoplasmic ratio of YAP signal in smooth muscle cells (lower histogram). Data are presented as mean ± SEM. *n* = 6–8 Data were analyzed by 1-way ANOVA followed by Tukey’s multiple comparisons test. NTG, non-transgenic; TnT-R92Q, transgenic mice expressing TnT-R92Q; TnI-DD, transgenic mice expressing TnI-S23,24D; DTG, double transgenic.

We have recently reported that YAP expression and localization transiently change in the coronary endothelium during HCM development in TnT-R92Q mice (Langa et al., 2023). Therefore, we tested whether these changes could be prevented by desensitization of myofilaments to Ca^2+^. Figure 8A shows that total YAP abundance and phosphorylation measured in the whole heart homogenates were not different between groups. Representative immunohistochemical images of transverse heart sections are presented in Figure 8B. Sections were stained with fluorescence antibodies against CD31, YAP, α-SMA, and DAPI to detect endothelial cells of cardiac vessels, YAP, smooth muscle cells, and the nucleus. Cytosolic and nuclear YAP signaling and their ratios in endothelial and smooth muscle cells are presented in Figures 8C,D, respectively. We found increased nuclear YAP signal and ratio of nuclear to cytosolic signals in endothelial cells from TnT-R92Q mice compared to NTG, but these changes were normalized in DTG hearts (Figure 8C). The ratio of nuclear to cytosolic signals was reduced in TnI-DD and DTG hearts compared to NTG (Figure 8D) but not significantly different in TnT-R92Q mice.

### 3.6 Fibrosis is increased in TnT-R92Q mice but was normalized by TnI-DD expression

**FIGURE 9:**
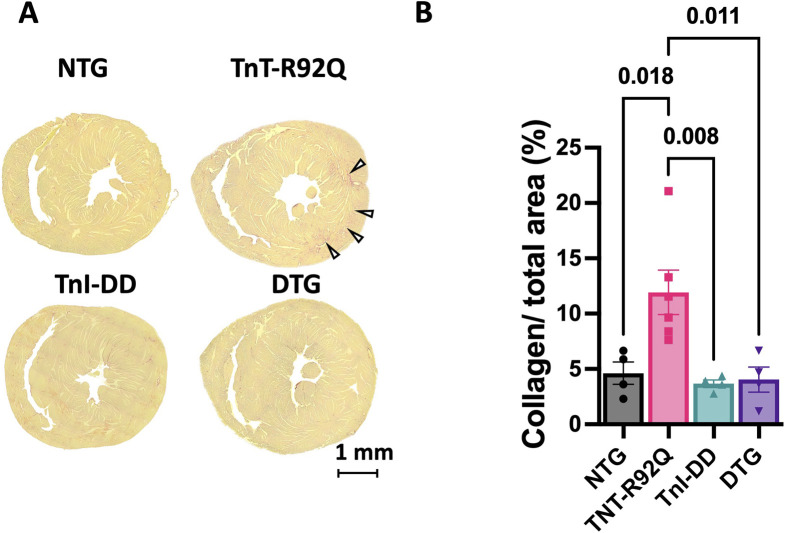
Fibrosis in midventricular regions of mouse hearts. **(A)** Representative trichrome-stained midpapillary images of NTG, TnT-R92Q, TnI-DD, and DTG hearts. **(B)** Quantitation of collagen deposition presented as % of covered area. Data are presented as mean ± SEM. *n* = 4–6. The normality of the data was tested using the Shapiro-Wilk test. Data were analyzed by 1-way ANOVA followed by Tukey’s test. NTG, non-transgenic; TnT-R92Q, transgenic mice expressing TnT-R92Q; TnI-DD, transgenic mice expressing TnI-S23,24D; DTG, double transgenic.

Figure 9A shows the representative trichrome-stained mid-papillary cross-sections of NTG, TnT-R92Q, TnI-DD, and DTG hearts. The quantification of collagen deposition is presented as a percentage of the total area and is presented in Figure 9B. Collagen expression is significantly increased in TnT-R92Q mice compared to other groups, but was normalized by TnI-DD expression.

### 3.7 Cardiac morphology and diastolic function stay improved in 16-week-old TnT-R92Q mice expressing TnI-DD

Finally, we also performed echocardiography in 16-week-old cohort mice to evaluate the long-term beneficial effects of TnI-DD expression in TnT-R92Q mice on cardiac morphology and function (Supplementary Table S4). Supplementary Figures S5A,B shows that further remodeling of TnT-R92Q hearts occurs at this time (increased LA size and LV mass). LA size was increased in TnT-R92Q compared to NTG but did not progress in DTG compared to TnT-R92Q hearts (Supplementary Figure S5A). LV mass was increased in TnT-R92Q mice, with no difference observed between NTG and DTG mice (Supplementary Figure S5B). We also observed no differences in LVID_d_ and RWT between groups (Supplementary Figures S5C,D). Moreover, no depression in systolic function was observed between NTG and TnT-R92Q groups (Supplementary Figures S5E,F), and no differences in CO, HR, and SV between groups (Supplementary Figures S5G–I). Similar to our previous report (Chowdhury et al., 2020), relaxation was further impaired in 16-week-old TnT-R92Q compared to NTG mice but significantly improved in DTG hearts (Supplementary Figures S5L–O). The only relaxation parameter different in the DTG heart was a slightly reduced A wave (Supplementary Figure S5L).

## 4 Discussion

Coordinating contractile function and coronary blood flow requires the interaction of cellular function among different cell types. Mutations in sarcomeric proteins that give rise to cardiomyopathies produce abnormalities in the biophysical properties of the sarcomere that are propagated beyond the cardiac myocyte. Understanding the progression of HCM disease demands a deeper understanding of how the loss of homogeneous function affects the heterogeneous population of cells within the heart. Experiments reported here demonstrate that expression of pseudo-phosphorylated cardiac TnI in the TnT-R92Q HCM mouse model results in partial normalization of myofilament Ca^2+^ sensitivity, improved cardiac morphology and function, reduced fibrosis, but a lack of normalization of coronary flow parameters. The novelty of the approach reported here highlights that although small corrections made to offset the sarcomeric defect may not fully or immediately resolve the pathophysiologic state of the disease, it can serve to lessen the severity of HCM.

Diastolic dysfunction was previously reported in HCM mutation-positive patients without cardiac hypertrophy (Ho et al., 2009; Norrish et al., 2024). Atrial size and diastolic dysfunction are good predictors of adverse events in pediatric patients (Nguyen et al., 2024). Several cellular mechanisms can contribute to diastolic dysfunction and should be considered as some potential new targets for developing new treatments. These include increased myofilament Ca^2+^ sensitivity, altered kinetics or distribution of cross-bridges, fibrosis, and altered Ca^2+^ fluxes. Coutu et al. (2004) have reported that delayed relaxation in Tm-A62V and Tm-E180G HCM models can be corrected by facilitating the Ca^2+^ sequestration process by overexpressing parvalbumin. We have previously reported improved phenotype and function in mouse models with HCM mutation in Tm (Tm-E180G) and TnT-R92Q by manipulating Ca^2+^ fluxes (Pena et al., 2010; Gaffin et al., 2011; Chowdhury et al., 2020). The protective effect was observed for at least 1 year in Tm-E180G-PLNKO (Gaffin et al., 2011) and TnT-R92Q-PLNKO (data not published) mice. Interestingly, PLNKO in TnT-R92Q mice did not change myofilament Ca^2+^ sensitivity, suggesting that myofilament sensitivity *per se* is not required for phenotype improvement in HCM. Davis et al. (2016) demonstrated that in genetically linked cardiomyopathies, twitch-time integral predicts the type of cardiac growth and severity of remodeling. We think manipulating Ca^2+^ fluxes improves the twitch-time integral, delaying the development of the HCM phenotype. Therefore, we can speculate that any interventions that normalize the twitch-time interval can potentially benefit the HCM phenotype. Our previously published (Alves et al., 2014) and current data clearly show that even a small shift in myofilament Ca^2+^ sensitivity in HCM linked to thin filament mutations toward typical values may be sufficient to delay the development of HCM. In addition to the myofilament desensitization, we have observed increased phosphorylation in PLN at Ser-16. It was reported that phosphorylation of Ser-16 mediates the maximal cardiac response to β1-adrenergic stimulation (Chu et al., 2000) and improves relaxation. Therefore, in our DTG hearts, twitch-time interval and cardiac relaxation are most likely improved due to improved Ca^2+^ transient decay and reduced myofilament Ca^2+^ sensitivity. Recently, Powers et al. (2020) demonstrated that modulating the tension-time integral of the cardiac twitch prevents dilated cardiomyopathy in the Tm-D230N DCM mouse model. Moreover, our data suggest that gene replacement therapy or allele-specific RNAi may be therapeutic (Jiang et al., 2013) since even small desensitization is effective.

The desensitization of myofilaments to Ca^2+^ in DTG mice was reduced, most likely by compensatory changes in myofilament protein expression and their modifications. We found that the expression of TnI-DD in TnT-R92Q mice resulted in an increased expression of TnT-R92Q in DTG mice compared to TnT-R92Q mice (Figure 4A), which diminishes the desensitization. Interestingly, the expression of TnI-DD in Tm-E180G mice did not alter the expression of mutated Tm and resulted in similar myofilament desensitization (Alves et al., 2014). The desensitization of myofilaments to Ca^2+^ in DTG mice was also most likely reduced by increased total phosphorylation of RLC in DTG mice compared to TnT-R92Q (Figure 5F). However, this increase in RLC phosphorylation between DTG and TnT-R92Q mice did not reach statistical significance when we used Phos-Tag gels (Supplementary Figure S3). Moreover, our data show decreased expression of TnT and TnI in TnT-R92Q mice that were partially rescued by expression of TnI-DD (Figure 4). We postulate that the decreased TnT and TnI expression in the mutant TnT-R92Q may reflect inefficient incorporation into the myofilaments, resulting in myofibrillar disarray and increased degradation via the ubiquitin-proteasome pathway.

Several limitations exist in using TnI-DD expression to desensitize the myofilaments to Ca^2+^. One of the limitations of pseudo-phosphorylation of TnI is that these modifications are permanent modifications of TnI. In contrast, phosphorylation is transient and reversible, and the phosphorylation levels can be regulated. During β-adrenergic stimulation, phosphorylation of both PLN and TnI at Ser 23,24 contributes to the enhanced relaxation rate (Kranias and Solaro, 1982; Wolska et al., 2002; Pena and Wolska, 2004). However, DTG mice lack this critical TnI contribution to enhance relaxation. Moreover, it is also possible that pseudo-phosphorylation of TnI at Ser 23, 24 alters other sites’ phosphorylation on TnI and further modulates sarcomere properties (for review, see (Biesiadecki and Westfall, 2019)). Another concern is the potential uncoupling of myofilament Ca^2+^ sensitivity from TnI phosphorylation in genetically linked cardiomyopathies. *In vitro* motility assay studies demonstrated that there is uncoupling between TnI phosphorylation and myofilament Ca^2+^ sensitivity, and this uncoupling was reversed by epigallocatechin-3-gallate (EGCG) (Papadaki et al., 2015; Messer et al., 2016). Usage of epigallocatechin-3-gallate indicates that the TnI phosphorylation uncoupling is reversible and does not require the removal of the initial trigger (HCM mutation). Recently, Yang et al. reported that silybin B, resveratrol, and EGCG restore the phosphorylation-dependent modulation of myofilament Ca^2+^-sensitivity in preparations with HCM and DCM mutations, resulting in improved lusitropy (Yang et al., 2024). Moreover, Sequeira et al. (2013) conducted studies in cardiac samples from patients with HCM, harboring mutations in MYH7, MYBPC3, TNNT2, TNNI3, and TPM1 filament proteins. They reported higher myofilament Ca^2+^ sensitivity when compared to sarcomere mutation negative HCM and non-failing donors, which correlated with low phosphorylation of PKA targets compared with those of donors. However, after exogenous PKA treatment, myofilament Ca^2+^ sensitivity decreased except for one preparation, indicating a lack of uncoupling of myofilament Ca^2+^ sensitivity from TnI phosphorylation. These data suggest that the uncoupling of myofilament Ca^2+^ sensitivity from TnI phosphorylation in HCM may depend on the mutation and the loading conditions of the myofilaments.

Data presented here show that the level of fibrosis was reduced to control levels in 28-day-old DTG mice. This reduction of fibrosis may contribute to the improvement of diastolic function in DTG mice. Both interstitial and replacement myocardial fibrosis have been observed in HCM patients. Ellims et al. (2012) reported that diffuse myocardial fibrosis in HCM patients is associated with LV diastolic dysfunction. Using late gadolinium enhancement cardiovascular NMR, Zhi et al. (2024) recently reported the presence of focal ischemic myocardial fibrosis in patients with HCM. Dense replacement fibrosis has been observed in almost 50% of children and adolescents with overt HCM and has progressed over time (Axelsson Raja et al., 2018). Moreover, even before the development of LVH, HCM patients have altered serum PICP, a biomarker of collagen metabolism, and this profibrotic state preceded fibrosis that could be detected on MRI (Ho et al., 2010). Studies presented by Zhi et al. (2024) suggest that myocyte disarray is a result of a direct response to the altered myofilament properties, but fibrosis and small vessel disease are secondary, and no relation was found between disarray, fibrosis, and small vessel disease. We have recently reported that in TnT-R92Q mice, fibrosis was detected as early as 7-day-old mice and worsened over the next 3 weeks, which correlates well with the human data discussed above.

Similarly to Tm-E180G mice (Alves et al., 2014), morphological and functional cardiac parameters were improved by the expression of TnI-DD in TnT-R92Q mice, and the development of the HCM phenotype was delayed. In our previous studies by Alves et al., we focused on age-related changes in the development of HCM phenotype, but did not include any studies related to the changes in coronary function in HCM. Here, we focused on the early point of HCM development, 28-day-old mice, before compensation was developed. We found that at this early age, morphological parameters (HW, HW/BW, and LA) of hearts from DTG mice did not differ from NTG hearts. DTG mice have improved relaxation compared to TnT-R92Q mice but show the first signs of early diastolic dysfunction, which was manifested by prolonged IVRT, and there is no significant change in E/A ratio compared to NTG hearts. When DTG mice age, they develop more diastolic dysfunction, as seen by the increase in the E/A ratio and the absence of change in IVRT (Supplementary Table S4). It is important to emphasize that in 28-day-old mice, we found a reduction in HR in TnT-R92Q and DTG groups compared to NTG (Figure 1; Supplementary Table S1). At lower HR, filling time increases, which could result in an increased SV, EF, and diastolic function.

Our recently published data show that TnT-R92Q mice show diastolic dysfunction as early as 7-day-old mice, and these changes were associated with alterations in coronary flow dynamics (Langa et al., 2023). Coronary dysfunction and impairment in coronary flow reserve (CFR) in adult patients with HCM have been previously reported (Camici et al., 1991; Sciagra et al., 2017). Tadamura et al. reported regional heterogeneity of CFR in HCM in pediatric patients (Tadamura et al., 2000). We speculated that normalization of increased myofilament Ca^2+^ sensitivity and, as a result, diastolic function should also improve these alterations in coronary flow dynamics. However, our current data show that despite significant desensitization of myofilament to Ca^2+^ observed in DTG mice, the prolonged coronary diastolic acceleration time seen in TnT-R92Q mice was not improved in DTG mice (Figure 2A). Finding the mechanisms responsible for this lack of normalization of coronary diastolic acceleration time is beyond the project’s scope, but we speculate that normal coronary function would require full normalization of diastolic function, which did not happen in our DTG mouse model since IVRT was prolonged. We have previously observed that the expression of cytoplasmic YAP significantly increased in the endothelium of coronary arteries in TnT-R92Q mice at 14 days of age. This rise in cytoplasmic YAP led to a decrease in the nuclear-to-cytosolic YAP ratio. However, this ratio was reversed by 28 days of age, resulting in an increased nuclear-to-cytosolic YAP signal (Langa et al., 2023). Interestingly, small desensitization was sufficient to normalize YAP nuc/cytosolic ratio in EC (Figure 8C). YAP and the transcriptional co-activator with PDZ-binding motif (TAZ) constitute a terminal effector complex that transduces mechanical signals into genetic change. EC-specific deletion of YAP/TAZ results in apoptosis and vascular defects during embryonic development (Wang et al., 2017). Mechanisms switching ON or OFF the Hippo signaling influence the intracellular localization and protein stability of YAP/TAZ through phosphorylation by upstream kinases (e.g., MST1/2 and LATS1/2). Phosphorylation of YAP at different sites either sequesters it in the cytoplasm or primes it for ubiquitination and degradation, preventing its nuclear translocation and TEAD transcription factor association. Most studies investigating HIPPO signaling in the heart have focused on the cardiomyocyte (Yamamoto et al., 2003; Xin et al., 2011; Del Re et al., 2013; Artap et al., 2018). Our data suggest that turning ON the pathway (normalizing YAP nuc/cytosolic ratio) may represent a mechanism by which myofilament desensitization represses pro-angiogenic genes driven by nuclear YAP/TAZ, such as connective tissue growth factor (CTGF) and cysteine-rich angiogenic inducer 61 (CYR61), to normalize and stabilize vascular remodeling in the DTG hearts (Brigstock, 2002). Additionally, we have previously reported that the total expression of YAP was increased in 14-day-old TnT-R92Q mice, but this increase was transient (Langa et al., 2023). However, while we observed a trend towards increased YAP expression in EC in TnT-R92Q mice here and a significant increase in the YAP nuc/cytosolic ratio, both were reduced in DTG hearts (Figure 8C), again further indicating that the net effect of inside/out and outside/in mechanical stresses acting on EC was normalized. These data indicate a role of myofilament desensitization in the modulation of Hippo signaling in the cardiac compartment. This modulation is most likely cell-specific and complex, requiring further experiments. This concept is supported by a recent review by Hu et al. (2024), which emphasizes the complexity of Hippo signaling among the various cell types in the cardiac microenvironment.

Our current and previous (Alves et al., 2014) studies suggest that desensitization of myofilament Ca^2+^ sensitivity in HCM linked to thin filament mutations is a valid target for future drug development. Although mavacamten is approved for treating adult symptomatic obstructive HCM (Keam, 2022), and it has been shown in different experimental models that it can improve relaxation (Nag et al., 2023; Ma et al., 2024), it may not be effective in neonatal patients. We have tested the effect of mavacamten on Ca^2+^-activation of mature and immature mouse cardiac myofilaments and found that it decreased myofilament Ca^2+^ sensitivity in adult TG hearts expressing neonatal ssTnI, but in myofilaments from 7-day-old TG hearts expressing TnT-R92Q and ssTnT, decreased only max tension (Halas et al., 2022). No data are available for the treatment of pediatric HCM patients. However, the new clinical trial (Clinical trial NCT06253221) to study its effectiveness in adolescents (age 12–18) with symptomatic obstructive hypertrophic cardiomyopathy is currently in its initial recruiting phase.

In summary, our studies reveal that even small but early desensitization of myofilaments to Ca^2+^ in HCM linked to TnT-R92Q mutation resulted in an improvement of cardiac function observed up to 16 weeks of age, prevention of fibrosis, and normalization of YAP signaling in EC. Despite these benefits, the coronary flow dynamics were not normalized. Further studies are needed to better understand the interaction between multiple cell types and their role in disease development and potential additional new targets for treatment.

## Data Availability

The original contributions presented in the study are included in the article/Supplementary Material, further inquiries can be directed to the corresponding author.
