# Supplementary material for: Reduction in myofilament Ca2+ sensitivity partially ameliorates the cardiac phenotype in hypertrophic cardiomyopathy linked to a TnT-R92Q mutation
Source: Front Physiol. 2025 May 23;16:1600117. doi: 10.3389/fphys.2025.1600117 (PMC12141004; doi:10.3389/fphys.2025.1600117)
Supplement: Supplementary file 1 [file DataSheet1.pdf]

Supplementary Material

Reduction in Myofilament Ca^{2+} Sensitivity Partially Ameliorates the Cardiac Phenotype in Hypertrophic Cardiomyopathy Linked to a TnT-R92Q Mutation

Paulina Langa[1,3] Angelie Bacon[1], Chad M. Warren[1], Shamim K. Chowdhury[1], Monika Halas[1], Aurelia A. Fernandes[1], Mark D. McCauley[2,3], Paul H. Goldspink[1,3], R. John Solaro[1,3], Beata M. Wolska[1,2,3,#]

[1]Department of Physiology and Biophysics, College of Medicine, University of Illinois, Chicago

[2]Department of Medicine, Division of Cardiology, College of Medicine, University of Illinois, Chicago

[3]Center for Cardiovascular Research, College of Medicine, University of Illinois, Chicago

[#]Corresponding author

Beata M. Wolska, PhD

Department of Medicine, Division of Cardiology

University of Illinois

840 S Wood St. Rm. 1112 (M/C 715)

Chicago, IL 60612

Ph. 312-413-0240

Fax 312-996-5062

1. Supplementary Methods

SDS-PAGE and Immunoblotting

Excised heart samples were immediately frozen in liquid nitrogen and stored at -80°C. Heart samples (10-20 mg) were homogenized with a Bead Ruptor 24 Elite as previously described (Batra et al., 2021; Capote et al., 2021). The homogenized sample was split equally for whole homogenate and myofibril preparations. The myofibril preparation was prepared with 1% (v/v) Triton X-100 (Solaro et al., 1971). The pellet was washed with SRB without Triton X-100 and resuspended 1:5 relative to the original tissue weight in industrial-strength buffer (ISB: 8 M urea, 2 M thiourea, 50 mM Tris pH 6.8, 3% v/v SDS, 75 mM DTT, and 0.05% bromophenol blue (Fritz et al., 1989). The whole homogenate preparations were solubilized at 1:5 relative to the original tissue weight in the ISB buffer. Protein concentrations were determined with 660 nM Protein Assay (ThermoFisher, 22660) with IDCR reagent.

Whole homogenate protein samples were loaded (10-25 µg/lane) on 12 or 15% (w/v) total acrylamide SDS-PAGE gels, with 0.5% (w/v) bis-acrylamide as previously described (Fritz et al., 1989). The gels were cast in Bio-Rad's Criterion Cell for most experiments except for myosin heavy chain and regulatory light chain (RLC) separations described below. Myosin heavy chain isoform separation was carried out in 6% (w/v) total acrylamide SDS-PAGE as previously described (Warren and Greaser, 2003) and stained with Coomassie G-250 (Bio-Rad, 1610786). The RLC separations utilized Phos-tag SDS-PAGE as previously described, with minor modifications (Kinoshita et al., 2006), and with 4 µg/lane of myofibril heart sample loaded onto the gel. RLC was separated into multiple bands corresponding to unphosphorylated (U), one (P1), and two (P2) phosphorylation sites, all within the same lane, allowing simple ratio analysis. The Phos-tag gel was 12% (w/v) total acrylamide, 3.3% (w/v) bis-acrylamide, 50 µM Phos-tag, 100 µM MnCl, and poured into 1 mm-thick Bio-Rad mini gel glass plates. The gel was run in a Bio-Rad mini gel apparatus at 20 mA for 75 min at room temperature, and then the proteins were transferred to the immunoblot membrane.

The protein transfers were done as previously described with some modifications (Matsudaira, 1987). The proteins were transferred onto 0.2 µm polyvinylidene difluoride (PVDF) membrane in 10 mM CAPS pH 11.0 without methanol at 20-30 V for 90 min. The transfer of the Phos-tag gels required preincubation with 10 mM CAPS pH 11.0 and 5 mM EDTA for 10 min, repeated once, and then washed once in 10 mM CAPS pH 11.0 buffer before transferring at 30V for 90 min. After the transfer, the membranes were blocked with either 5% (w/v) non-fat dry milk (NFDM) in 50 mM Tris-HCl pH 7.5, 200 mM NaCl with 0.1% (v/v) Tween-20 (TBST) or 2% BSA-TBST. The immunoblots were incubated in primary antibodies overnight at 4°C, washed in TBST, incubated in secondary antibodies at room temperature for 1.5 hrs, and washed in TBST. See Supplemental Table 5 for the specific antibody information. The membranes were developed with ECL (ThermoFisher, 34096 or Bio-Rad, 170-5061), imaged with Chemidoc MP (Bio-Rad), and analyzed with ImageLab (Bio-Rad, v. 6.0.1). The data were statistically analyzed and graphed with GraphPad Prism v 9.3.1 or 10.0.3.

To determine overall phosphorylation levels of myofilament proteins, myofibril heart samples (7 µg/lane) were loaded onto 15% (w/v) total acrylamide SDS-PAGE. The gel was stained with a Pro-Q Diamond stain (Invitrogen, P33301) following the manufacturer's recommendations. The gel was imaged with Bio-Rad's Chemidoc MP imager, after which the gel was stained with Coomassie G-250 (Bio-Rad, 1610786) following the manufacturer's recommendations. The images were analyzed using Bio-Rad's Image Lab V 6.0.1 and Microsoft Excel 360. The data were statistically analyzed and graphed with GraphPad Prism v 9.3.1 or 10.0.3.

Echocardiography

B-mode, M-mode, pulsed-wave Doppler, and tissue Doppler images were obtained as previously described (Alves et al., 2014; Chowdhury et al., 2020) in four groups of animals. Mice were anesthetized with 3-4% isoflurane in an induction chamber, followed by maintenance at 1-3% isoflurane concentrations through a respirator. Body temperature was monitored by a rectal probe and maintained at 37°C. Electrode conduction gel was applied to the distal extremities, which were taped to electrodes. Upper abdominal and anterior chest wall hair was removed and cleaned away before applying acoustic conduction gel. The left atrial diameter was assessed by B-mode and M-mode images acquired in the parasternal long-axis window at the aortic root level. B-mode and M-mode images were used for multiple parasternal short-axis windows (apical, mid-ventricular, and basal), with the mid-ventricular/papillary level singled out for assessment of posterior and anterior wall thickness and ventricular luminal diameter during both systole and diastole to calculate fractional shortening, stroke volume, and cardiac output. The mice were then repositioned to the Trendelenburg position to obtain B-mode and pulse-wave Doppler images of the apical four-chamber window for mitral inflow measurements and tissue Doppler for septal mitral annular velocities. All measurements and calculations were averaged from three consecutive cycles and performed according to the American Society of Echocardiography guidelines. Data analysis was performed with the VevoLab 5.5.1. Analytic Software.

High-quality coronary flow velocity signals were obtained under isoflurane-induced anesthesia, as described above. The coronary vasodilator properties of isoflurane are well known, so we strictly controlled the level of isoflurane input and heart rate to ensure the accuracy of the collected data. Coronary flow measurements were performed on a modified parasternal long-axis view as previously described (Chang et al., 2015). From the low parasternal short-axis view, a search for diastolic color velocity in the anterior interventricular groove, followed by clockwise rotation to achieve alignment of the color jet, was performed. The sample volume position was consistent in all mice during the measurements.

Fibrosis Assessment

The deparaffinized sections were stained for collagen depositions (fibrosis) using the Picro Sirius Stain kit (Abcam, ab) according to the manufacturer's instructions. The Trichrome stain kit was intended for visualization of collagenous connective tissue fibers in tissue sections. Coverslips were mounted with Krystalon toluene-based mounting medium (Harleco, 64969-71). Next, images of whole heart sections were taken by a Zeiss Axio Imager Z2 (Germany) brightfield microscope with a motorized stage for tiling. Tiles (region of scanning) were fused using native Zen stitching. Analysis of fibrosis levels in whole heart scans of apex/apical and midventricular levels was done using ImageJ (NIH ver. 1.53k14) in heart sections. The Trichrome-stained fibrosis images were analyzed by taking the original RGB image color channels and selecting the color channel corresponding to the trichrome stain. The channel was then manually adjusted to the pixel threshold values that best fit the collagenous staining. The area was measured using ImageJ's Measure tool with the Limit to Threshold property enabled. The tissue/background was determined by minimal auto-thresholding of the same channel. The fraction of the collagenous area was calculated by dividing the collagenous area by the tissue area. Localized fibrosis was assessed by 2048 x 2048 square pixel window selection of regions of interest (coronary artery regions – CA, right ventricular insertion – RVI, intraventricular septum- IVS, lateral free wall- LW). Levels of fibrosis were measured as percent collagenous area to tissue area (within the scanned window).

Immunohistochemistry (IHC) and Histology

Mice were anesthetized with 5% isoflurane, and the hearts were excised and placed into cold PBS. The hearts were quickly sliced at the midpapillary level and placed into biopsy cassettes, followed by fixation in 10% neutral buffered formalin (Milipore-Sigma, HT501128), then washed and stored in 70% Ethanol. Next, samples were paraffin-embedded, and non-consecutive transverse sections were cut and applied to microscope slides (Research Histology Core, UIC). The formalin-fixed and paraffin-embedded sections were baked at 60°C and then deparaffinized with 100% xylene (2 x 7 min) followed by rehydration with incremental washes of decreasing aqueous ethanol (100% for 2 x 5 min, 95% for 5 min, 70% for 5 min, and 50% for 5 min) solutions, and washed in distilled water for 20 min and used for staining or continued on for IHC. Next, antigen retrieval was performed using sodium citrate buffer (10 mM sodium citrate, 0.05% Tween 20, pH 6.0) at 95°C for 40 min.

Sections were then blocked in 5% BSA in PBS Tween 20 (PBST) (0.1% Tween-20) for 1 hour at room temperature. To visualize vessels, sections were incubated in rat monoclonal anti-CD31 antibody (1:10, cat. DIA-310, Dianova) and α-SMA. To detect YAP, rabbit polyclonal anti-YAP (1:100) was used in 1% BSA TBST and incubated overnight at 4°C. Next, after three 5-minute washes with PBST, sections were incubated with secondary antibodies for 2 hours at room temperature. Next, sections were washed three times for 5 min and incubated with DAPI (4',6-diamidino-2-phenylindole) for nuclear counterstaining for 20 min at room temperature. Sections were then washed in TBST and mounted with a mounting medium preserving fluorescent signal (ThermoFisher Scientific, P10144). For a negative primary antibody (NPA) control, we omitted the primary antibodies. All sections were airy-scanned at 16-bit values in the regions of interest with a Zeiss LSM880 confocal microscope (Germany). The camera used for acquisition had a single GaAsP photomultiplier tube (PMT), and the light was filtered by emission filters (EF5) with acoustic-optical tunable filters (AOTF) to adjust the necessary brightness.

All slides were scanned at 1024 x 1024-pixel size with 16-bit depth values with an objective C-apochromatic 63x/1.2 W Korr FCS M27. Channels with their properties included: (1) Channel 1 (633nm) with gain of 800, and ILP (illumination power) 1.50%; (2) Channel 2 (561 nm) with gain of 800, and ILP (illumination power) 4.00%; (3) Channel 3 (488 nm) with gain of 850, and ILP (illumination power) 4.00%; and (4) Channel 4 (405 nm) with gain of 750, and ILP (illumination power) 1.00%. Images were acquired using ZEISS Black 2.3 SP1 software and analyzed using ZEISS Blue edition 3.2 software.

YAP signal intensity measurement in coronary vessels.

Acquired images were then uploaded to Image J ver. 1.54 for fluorescence intensity measurements. Three non-consecutive slides per animal in each group were analyzed. An area of coronary vessels was outlined, and a signal intensity corresponding to YAP mean fluorescence expressed in pixels was collected. The values were plotted onto graphs, and statistical analysis was performed (see details in Statistical analysis).

2. **Supplementary Data**

   a. **Supplementary Figures**

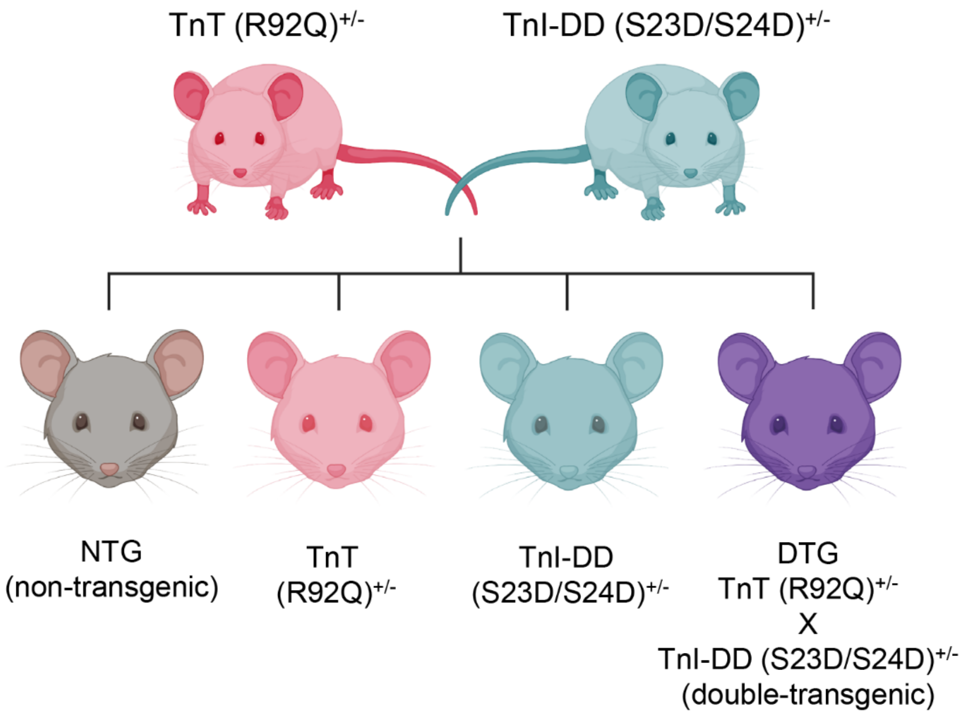

Supplemental Figure 1. The schematic representation of breeding the mice and the generation of experimental groups. NTG, non-transgenic; TnT-R92Q - transgenic mice expressing TnT-R92Q, TnI-DD – transgenic mice expressing TnI-S23,24D, DTG - double transgenic.

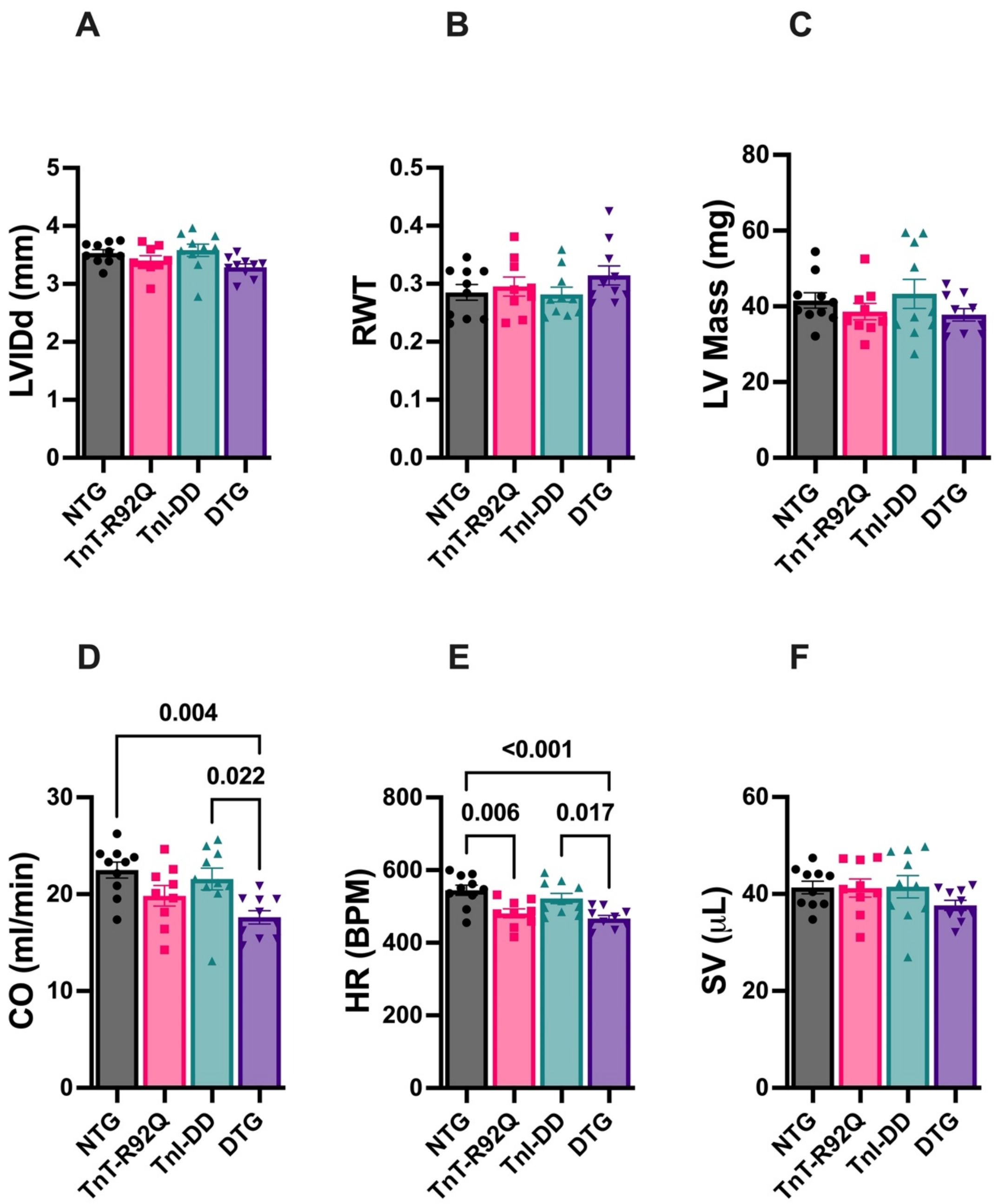

Supplemental Figure 2. Morphological, systolic, and diastolic parameters in NTG, TnT-R92Q, TnI-DD, and DTG hearts at 28 days of age. **(A)** left ventricular internal diastolic diameter (LVIDd, **(B)** relative wall thickness (RWT), **(C)** left ventricular mass calculated based on echocardiography (LV Mass), **(D)** cardiac output (CO), **(E)** heart rate (HR), **(F)** stroke volume (SV). Data are presented as mean ± SEM. n=9-10; Data were analyzed by 1-way ANOVA followed by Tukey's multiple comparisons test. NTG, non-transgenic; TnT-R92Q - transgenic mice expressing TnT-R92Q, TnI-DD – transgenic mice expressing TnI-S23,24D, DTG - double transgenic.

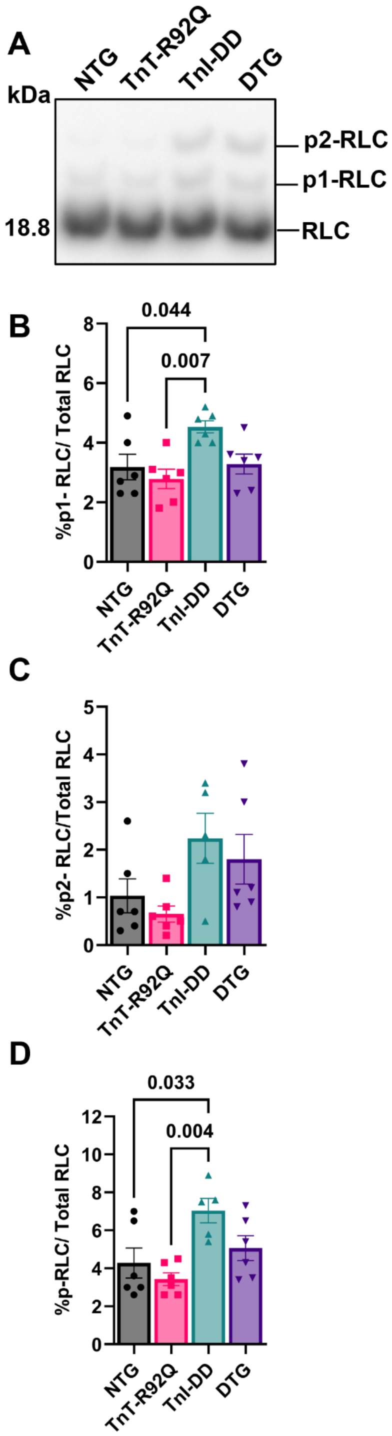

Supplemental Figure 3. **Phosphorylation (p) of the regulatory light chain (RLC) in isolated myofilaments via Western blot PhosTag separation**. **(A)** Representative Western blot PhosTag image of regulatory light chain. **(B)** Quantitation of p1-RLC site compared to total RLC abundance. **(C)** Histogram of p2-RLC site compared to total RLC abundance. **(D)** Histogram of all p-RLC sites compared to total RLC abundance. Data reported as mean ± SEM, n=5-6. Data were analyzed by 1-way ANOVA followed by Tukey's test. NTG, non-transgenic; TnT-R92Q - transgenic mice expressing TnT-R92Q, TnI-DD – transgenic mice expressing TnI-S23,24D, DTG - double transgenic.

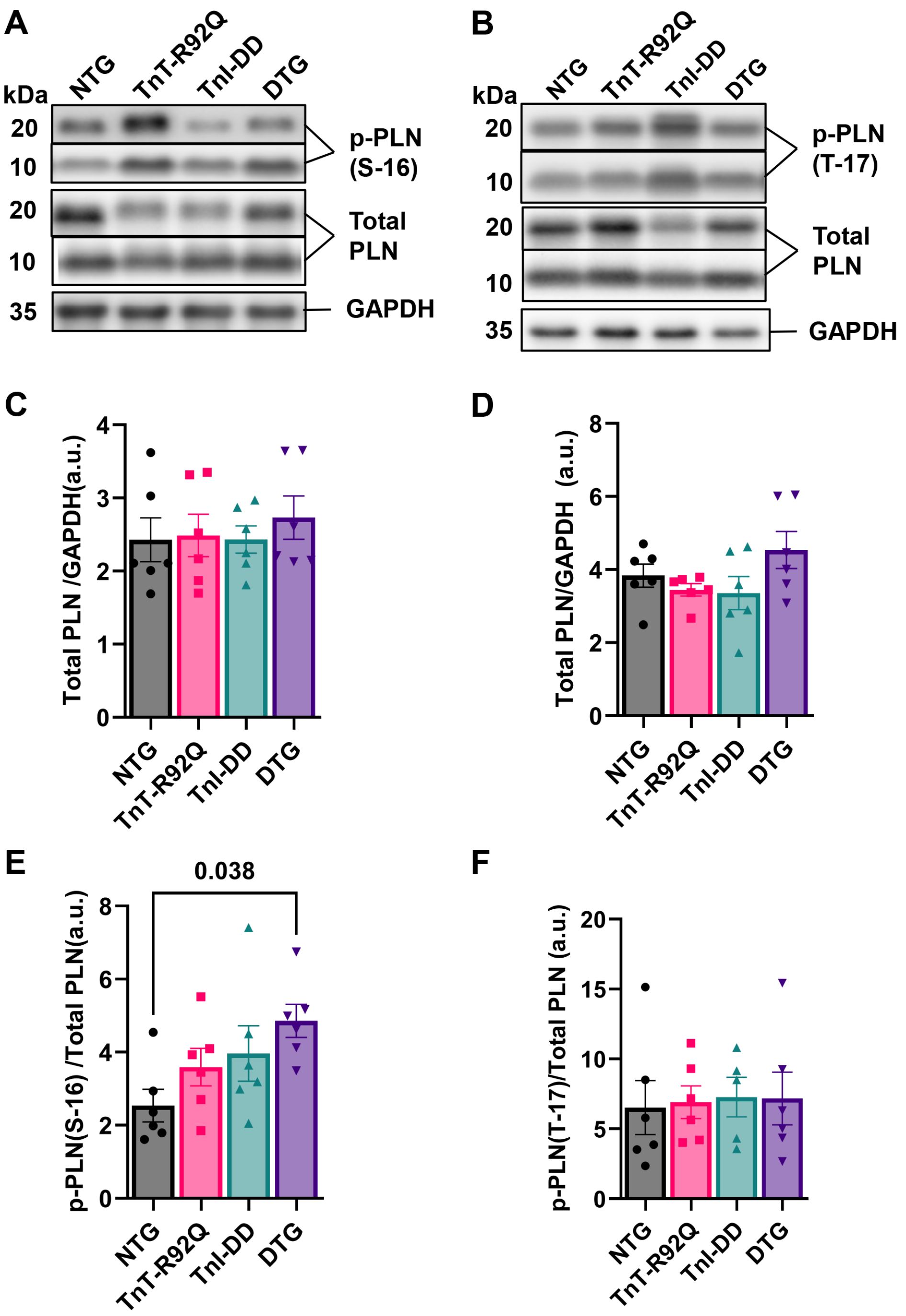

Supplemental Figure 4. Total phospholamban (PLN) abundance and phosphorylation (p) in whole heart homogenates. **(A)** Representative Western blot images of p-PLN at serine 16, Total PLN, and GAPDH loading control. **(B)** Representative Western blot images of p-PLN at threonine 17, Total PLN, and GAPDH loading control. **(C)** Histogram of total PLN/GAPDH abundance. **(D)** Histogram of total PLN/GAPDH abundance. **(E)** Histogram of p-PLN (S-16)/total PLN abundance. **(F)** Histogram of p-PLN (T17)/total PLN abundance. Data reported as mean ± SEM, n=5-6. Data were analyzed by 1-way ANOVA followed by Tukey's test. NTG, non-transgenic; TnT-R92Q - transgenic mice expressing TnT-R92Q, TnI-DD – transgenic mice expressing TnI-S23,24D, DTG - double transgenic.

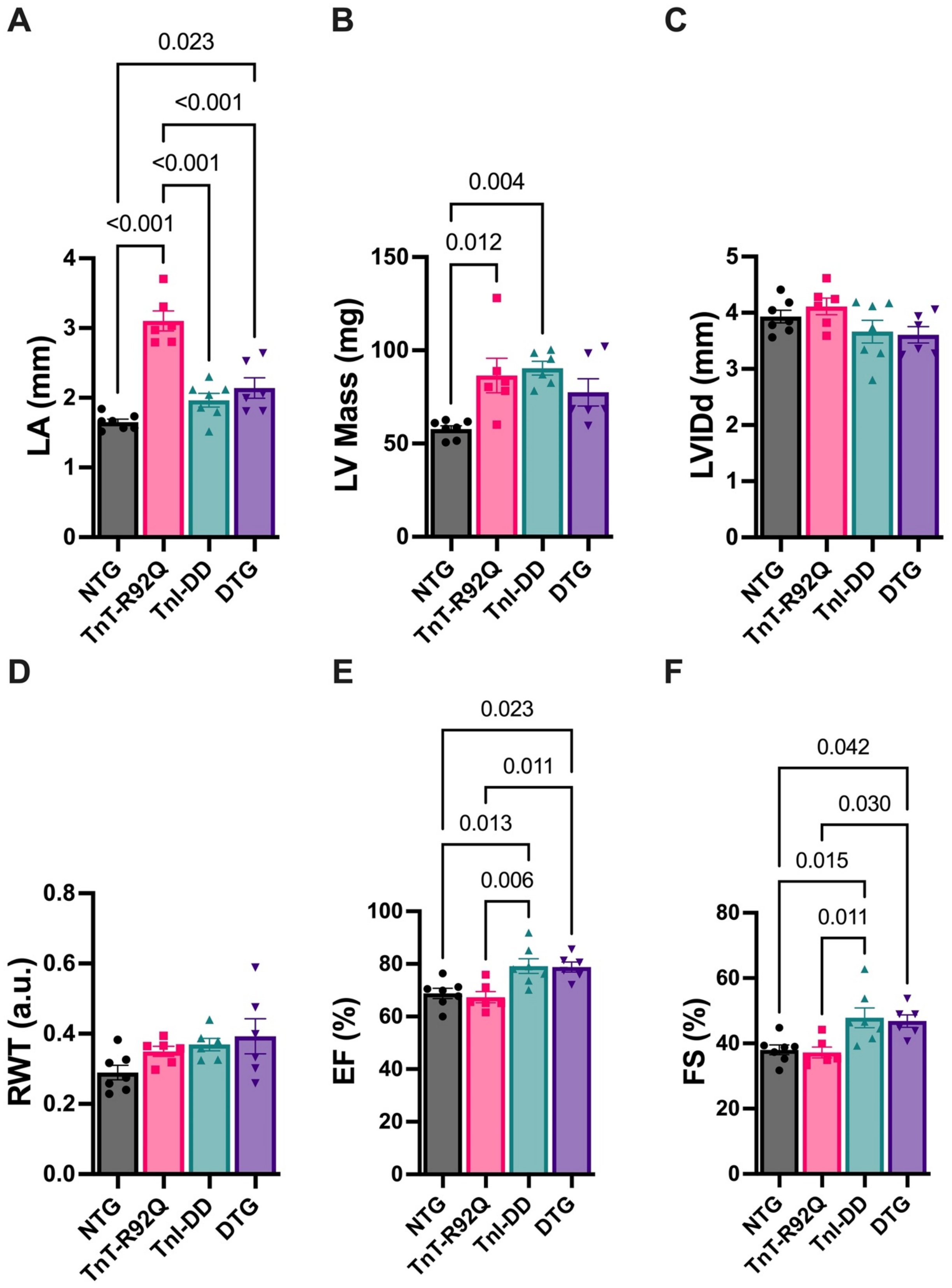

A
0.023
<0.001
<0.001
<0.001
LA (mm)
0
1
2
3
4
NTG
TnT-R92Q
TnI-DD
DTG
B
0.004
0.012
LV Mass (mg)
0
50
100
150
NTG
TnT-R92Q
TnI-DD
DTG
C
LVIDd (mm)
0
1
2
3
4
5
NTG
TnT-R92Q
TnI-DD
DTG
D
RWT (a.u.)
0.0
0.2
0.4
0.6
0.8
NTG
TnT-R92Q
TnI-DD
DTG
E
0.023
0.011
0.013
0.006
EF (%)
0
20
40
60
80
100
NTG
TnT-R92Q
TnI-DD
DTG
F
0.042
0.030
0.015
0.011
FS (%)
0
20
40
60
80
NTG
TnT-R92Q
TnI-DD
DTG

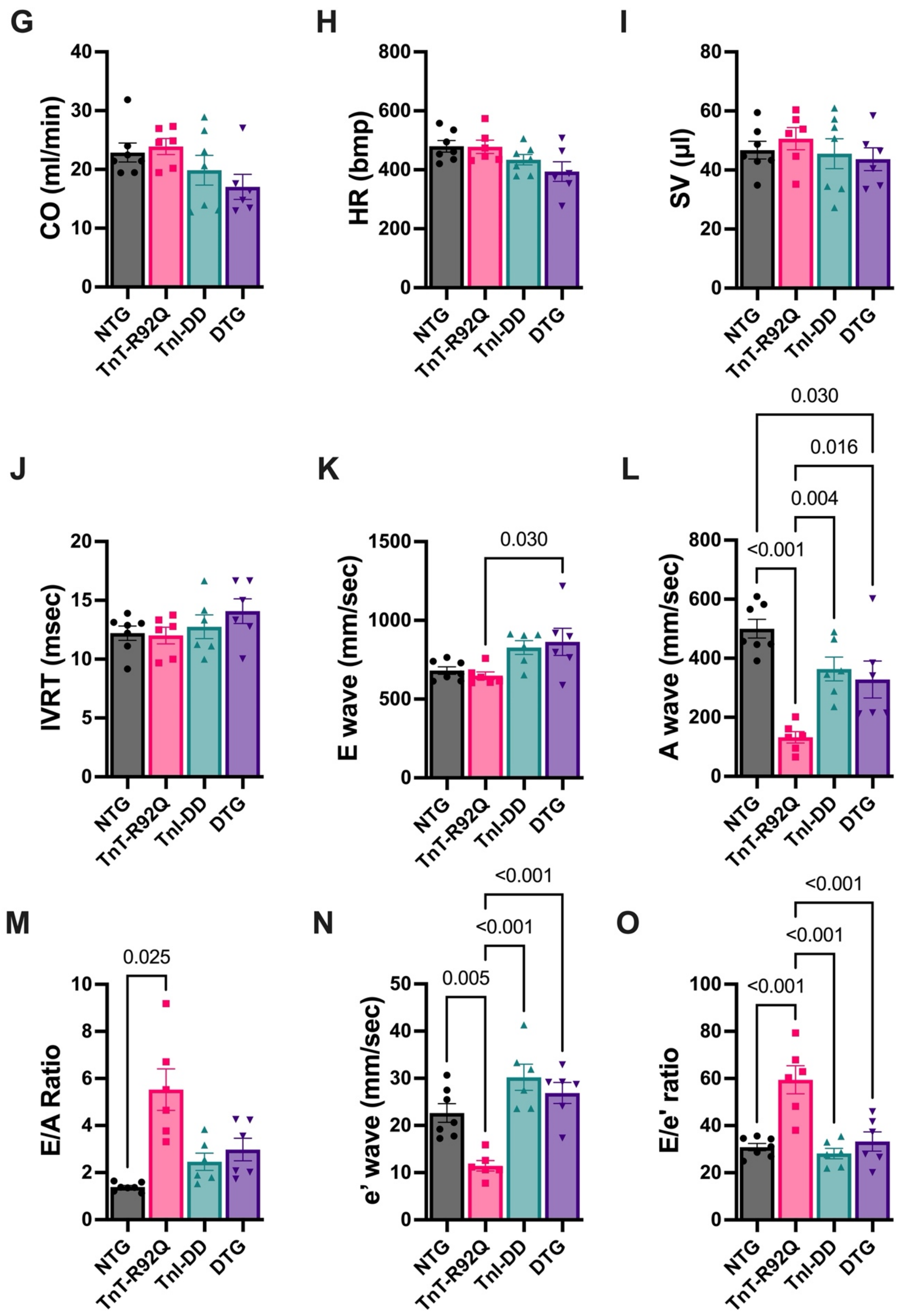
G
H
I
CO (ml/min)
HR (bmp)
SV (µl)
J
K
L
IVRT (msec)
E wave (mm/sec)
A wave (mm/sec)
0.030
0.030
0.016
0.004
<0.001
M
N
O
E/A Ratio
e' wave (mm/sec)
E/e' ratio
0.025
<0.001
<0.001
0.005
<0.001
<0.001
<0.001
NTG
TnT-R92Q
TnI-DD
DTG

Supplemental Figure 5. Morphological, systolic, and diastolic parameters in NTG, TnT-R92Q, TnI-DD, and DTG hearts at 16 weeks of age. (A) left atrial diameter (LA), **(B)** left ventricular mass calculated based on echocardiography (LV mass), **(C)** left ventricular internal diastolic diameter (LVIDd, **(D)** relative wall thickness (RWT), **(E)** ejection fraction (EF), **(F)** fractional shortening (FS), **(G)** cardiac output (CO), **(H)** heart rate (HR), **(I)** stroke volume (SV), **(J)** isovolumic relaxation time (IVRT), **(K)** peak velocity of early diastolic mitral flow (E) wave, **(L)** peak velocity of late diastolic mitral inflow A wave, **(M)** E/A ratio represents peak velocity of early diastolic mitral flow divided by peak velocity of late diastolic mitral inflow, **(N)** peak velocity of early diastolic mitral annual motion **(O)** E/e' ratio represents peak velocity of early diastolic transmitral flow divided by peak velocity of early diastolic mitral annual motion. Data are presented as mean ± SEM. n=6-7 Data were analyzed by 1-way ANOVA followed by Tukey's multiple comparisons test (panels A-C, E-L, N-O). RWT and E/A ratio data were analyzed using 1-way ANOVA, followed by Dunnett's T3 multiple comparisons test. NTG, non-transgenic; TnT-R92Q - transgenic mice expressing TnT-R92Q, TnI-DD – transgenic mice expressing TnI-S23,24D, DTG - double transgenic.

Supplemental Table 1. Morphological, Systolic, and Diastolic Parameters Evaluated by Echocardiography at 28 Days of Age.

<table>
<tr><th>Parameter</th><th></th><th>NTG
(n=10)</th><th>TnT-R92Q
(n=9)</th><th>TnI-DD
(n=10)</th><th>DTG
(n=10)</th></tr>
<tr><td rowspan="5">LA
(mm)</td><td>Mean</td><td>1.52</td><td>2.12</td><td>1.53</td><td>1.64</td></tr>
<tr><td>SE</td><td>0.058</td><td>0.059</td><td>0.084</td><td>0.054</td></tr>
<tr><td colspan="5">ANOVA P<0.001</td></tr>
<tr><td colspan="3">Tukey's multiple comparisons test</td><td colspan="2">Adjusted P Value</td></tr>
<tr><td colspan="3">NTG vs. TnT-R92Q
NTG vs. TnI-DD
NTG vs. DTG
TnT-R92Q vs. TnI-DD
TnT-R92Q vs. DTG
TnI-DD vs. DTG</td><td colspan="2"><0.001
>0.999
0.536
<0.001
<0.001
0.579</td></tr>
<tr><td rowspan="6">LV mass
(mg)</td><td></td><td>NTG
(n=10)</td><td>TnT-R92Q
(n=9)</td><td>TnI-DD
(n=10)</td><td>DTG
(n=10)</td></tr>
<tr><td>Mean</td><td>41.54</td><td>38.63</td><td>43.30</td><td>37.78</td></tr>
<tr><td>SE</td><td>2.040</td><td>2.174</td><td>3.810</td><td>1.617</td></tr>
<tr><td colspan="5">ANOVA P=0.404</td></tr>
<tr><td colspan="3">Tukey's multiple comparisons test</td><td colspan="2">Adjusted P Value</td></tr>
<tr><td colspan="3">NTG vs. TnT-R92Q
NTG vs. TnI-DD
NTG vs. DTG
TnT-R92Q vs. TnI-DD
TnT-R92Q vs. DTG
TnI-DD vs. DTG</td><td colspan="2">0.4360
0.6276
0.3021
0.2144
0.8183
0.1333</td></tr>
<tr><td rowspan="6">LVIDd
(mm)</td><td></td><td>NTG
(n=10)</td><td>TnT-R92Q
(n=9)</td><td>TnI-DD
(n=10)</td><td>DTG
(n=10)</td></tr>
<tr><td>Mean</td><td>3.53</td><td>3.40</td><td>3.58</td><td>3.29</td></tr>
<tr><td>SE</td><td>0.06</td><td>0.08</td><td>0.11</td><td>0.06</td></tr>
<tr><td colspan="5">ANOVA P=0.047</td></tr>
<tr><td colspan="3">Tukey's multiple comparisons test</td><td colspan="2">Adjusted P Value</td></tr>
<tr><td colspan="3">NTG vs. TnT-R92Q
NTG vs. TnI-DD
NTG vs. DTG
TnT-R92Q vs. TnI-DD
TnT-R92Q vs. DTG
TnI-DD vs. DTG</td><td colspan="2">0.650
0.972
0.129
0.396
0.738
0.051</td></tr>
<tr><td rowspan="6">LVISd
(mm)</td><td></td><td>NTG
(n=10)</td><td>TnT-R92Q
(n=9)</td><td>TnI-DD
(n=10)</td><td>DTG
(n=10)</td></tr>
<tr><td>Mean</td><td>1.93</td><td>1.68</td><td>2.02</td><td>1.48</td></tr>
<tr><td>SE</td><td>0.06</td><td>0.12</td><td>0.09</td><td>0.05</td></tr>
<tr><td colspan="5">ANOVA P<0.001</td></tr>
<tr><td colspan="3">Tukey's multiple comparisons test</td><td colspan="2">Adjusted P Value</td></tr>
<tr><td colspan="3">NTG vs. TnT-R92Q
NTG vs. TnI-DD
NTG vs. DTG
TnT-R92Q vs. TnI-DD
TnT-R92Q vs. DTG
TnI-DD vs. DTG</td><td colspan="2">0.1868
0.8504
0.0033
0.0357
0.3528
0.0004</td></tr>
</table>

RWT

	**NTG** (n=10)	**TnT-R92Q** (n=9)	**TnI-DD** (n=10)	**DTG** (n=10)
**Mean**	0.29	0.30	0.28	0.31
**SE**	0.014	0.016	0.013	0.017
ANOVA P=0.401				

Tukey's multiple comparisons test	Individual P Value
NTG vs. TnT-R92Q	0.963
NTG vs. TnI-DD	0.998
NTG vs. DTG	0.498
TnT-R92Q vs. TnI-DD	0.919
TnT-R92Q vs. DTG	0.805
TnI-DD vs. DTG	0.403

EF (%)

	**NTG** (n=10)	**TnT-R92Q** (n=9)	**TnI-DD** (n=10)	**DTG** (n=10)
**Mean**	75.15	83.40	74.15	86.80
**SE**	0.618	1.898	1.622	1.686
ANOVA P<0.001				

Tukey's multiple comparisons test	Adjusted P Value
NTG vs. TnT-R92Q	0.005
NTG vs. TnI-DD	0.968
NTG vs. DTG	<0.001
TnT-R92Q vs. TnI-DD	<0.001
TnT-R92Q vs. DTG	0.421
TnI-DD vs. DTG	<0.001

FS (%)

	**NTG** (n=9)	**TnT-R92Q** (n=9)	**TnI-DD** (n=10)	**DTG** (n=10)
**Mean**	43.18	52.07	42.50	56.49
**SE**	0.556	2.323	1.469	2.680
ANOVA P<0.001				

Tukey's multiple comparisons test	Adjusted P Value
NTG vs. TnT-R92Q	0.019
NTG vs. TnI-DD	0.995
NTG vs. DTG	<0.001
TnT-R92Q vs. TnI-DD	0.008
TnT-R92Q vs. DTG	0.401
TnI-DD vs. DTG	<0.001

CO (ml/min)

	**NTG** (n=10)	**TnT-R92Q** (n=9)	**TnI-DD** (n=10)	**DTG** (n=10)
**Mean**	22.50	19.83	21.57	17.62
**SE**	0.819	1.066	1.124	0.682
ANOVA P=0.004				

Tukey's multiple comparisons test	Adjusted P Value
NTG vs. TnT-R92Q	0.212
NTG vs. TnI-DD	0.894
NTG vs. DTG	0.004
TnT-R92Q vs. TnI-DD	0.569
TnT-R92Q vs. DTG	0.363
TnI-DD vs. DTG	0.022

**HR (bpm)**		**NTG** (n=10)	**TnT-R92Q** (n=9)	**TnI-DD** (n=10)	**DTG** (n=10)
	**Mean**	544.7	480.8	521.9	466.9
	**SE**	14.16	12.47	14.02	8.48
	ANOVA P<0.001				
	Tukey's multiple comparisons test		Adjusted P Value		
	NTG vs. TnT-R92Q		0.006		
	NTG vs. TnI-DD		0.567		
	NTG vs. DTG		<0.001		
	TnT-R92Q vs. TnI-DD		0.120		
	TnT-R92Q vs. DTG		0.866		
	TnI-DD vs. DTG		0.017		

**SV (μl)**		**NTG** (n=10)	**TnT-R92Q** (n=9)	**TnI-DD** (n=10)	**DTG** (n=10)
	**Mean**	41.35	41.21	41.50	37.66
	**SE**	1.29	1.86	2.31	1.04
	ANOVA P=0.317				
	Tukey's multiple comparisons test		Individual P Value		
	NTG vs. TnT-R92Q		>0.999		
	NTG vs. TnI-DD		>0.999		
	NTG vs. DTG		0.412		
	TnT-R92Q vs. TnI-DD		>0.999		
	TnT-R92Q vs. DTG		0.471		
	TnI-DD vs. DTG		0.378		

**IVRT (msec)**		**NTG** (n=10)	**TnT-R92Q** (n=9)	**TnI-DD** (n=10)	**DTG** (n=10)
	**Mean**	10.86	12.28	9.70	13.56
	**SE**	0.377	0.364	0.464	0.320
	ANOVA P<0.001				
	Tukey's multiple comparisons test		Adjusted P Value		
	NTG vs. TnT-R92Q		0.067		
	NTG vs. TnI-DD		0.160		
	NTG vs. DTG		<0.001		
	TnT-R92Q vs. TnI-DD		<0.001		
	TnT-R92Q vs. DTG		0.116		
	TnI-DD vs. DTG		<0.001		

**E wave (mm/sec)**		**NTG** (n=10)	**TnT-R92Q** (n=9)	**TnI-DD** (n=10)	**DTG** (n=10)
	**Mean**	836.9	715.8	963.8	851.0
	**SE**	28.36	36.30	25.83	31.36
	ANOVA P<0.001				
	Tukey's multiple comparisons test		Adjusted P Value		
	NTG vs. TnT-R92Q		0.042		
	NTG vs. TnI-DD		0.025		
	NTG vs. DTG		0.987		
	TnT-R92Q vs. TnI-DD		<0.001		
	TnT-R92Q vs. DTG		0.019		
	TnI-DD vs. DTG		0.055		

**A wave (mm/sec)**		**NTG** (n=10)	**TnT-R92Q** (n=9)	**TnI-DD** (n=10)	**DTG** (n=10)
	**Mean**	666.1	469.5	552.1	592.0
	**SE**	27.45	46.25	24.06	26.17
	ANOVA P=0.001				
	Tukey's multiple comparisons test		Adjusted P Value		
	NTG vs. TnT-R92Q		<0.001		
	NTG vs. TnI-DD		0.061		
	NTG vs. DTG		0.342		
	TnT-R92Q vs. TnI-DD		0.274		
	TnT-R92Q vs. DTG		0.047		
	TnI-DD vs. DTG		0.799		

**E/A Ratio**		**NTG** (n=10)	**TnT-R92Q** (n=9)	**TnI-DD** (n=10)	**DTG** (n=10)
	Mean	1.26	1.61	1.79	1.46
	SE	0.026	0.119	0.127	0.071
	Kruskal-Wallis test P value <0.001				
	Dunn's multiple comparisons test		Adjusted P Value		
	NTG vs. TnT-R92Q		0.040		
	NTG vs. TnI-DD		<0.001		
	NTG vs. DTG		0.343		
	TnT-R92Q vs. TnI-DD		>0.999		
	TnT-R92Q vs. DTG		>0.999		
	TnI-DD vs. DTG		0.177		

**e' (mm/sec)**		**NTG** (n=10)	**TnT-R92Q** (n=9)	**TnI-DD** (n=10)	**DTG** (n=10)
	**Mean**	28.68	16.95	30.84	23.32
	**SE**	1.593	1.623	1.253	1.608
	ANOVA P<0.001				
	Tukey's multiple comparisons test		Adjusted P Value		
	NTG vs. TnT-R92Q		<0.001		
	NTG vs. TnI-DD		0.743		
	NTG vs. DTG		0.074		
	TnT-R92Q vs. TnI-DD		<0.001		
	TnT-R92Q vs. DTG		0.030		
	TnI-DD vs. DTG		0.006		

**E/e’**		**NTG** (n=10)	**TnT-R92Q** (n=9)	**TnI-DD** (n=10)	**DTG** (n=10)
	**Mean**	30.06	45.25	31.70	37.54
	**SE**	2.140	4.287	1.441	1.973
	ANOVA P<0.001				
	Tukey's multiple comparisons test		Adjusted P Value		
	NTG vs. TnT-R92Q		0.001		
	NTG vs. TnI-DD		0.968		
	NTG vs. DTG		0.182		
	TnT-R92Q vs. TnI-DD		0.004		
	TnT-R92Q vs. DTG		0.178		
	TnI-DD vs. DTG		0.382		

NTG, non-transgenic; TnT-R92Q - transgenic mice expressing TnT-R92Q, TnI-DD – transgenic mice expressing TnI-S23,24D, DTG - double transgenic. Data presented as mean ± SEM. n = Sample sizes.

LA = left atrium, LV mass = left ventricle mass, LVIDd = left ventricular internal diameter at diastole, LVISd = left ventricular internal diameter at systole, RWT = relative wall thickness, EF = ejection fraction, FS = fractional shortening, CO = cardiac output, HR = heart rate, SV = stroke volume, IVRT = isovolumic relaxation time, E wave = peak velocity of early diastolic transmitral flow, A wave = peak velocity of late diastolic transmitral flow, e' – peak velocity of early diastolic mitral annular motion.

Supplemental Table 2.

Coronary Flow Parameters Evaluated by Echocardiography at 28 Days of Age.

Parameter		NTG (n=10)	TnT-R92Q (n=8)	TnI-DD (n=10)	DTG (n=10)
**Diastolic AT (msec)**	**Mean**	17.20	23.37	16.97	22.35
	**SE**	1.188	0.932	0.642	0.996
	ANOVA P<0.001				
	Tukey's multiple comparisons test		Adjusted P Value		
	NTG vs. TnT-R92Q		<0.001		
	NTG vs. TnI-DD		0.998		
	NTG vs. DTG		0.003		
	TnT-R92Q vs. TnI-DD		<0.001		
	TnT-R92Q vs. DTG		0.889		
	TnI-DD vs. DTG		0.002		
**Mean Diastolic Velocity (mm/sec)**		**NTG** (n=10)	**TnT-R92Q** (n=8)	**TnI-DD** (n=10)	**DTG** (n=10)
	**Mean**	380.7	314.6	433.0	352.6
	**SE**	34.94	9.830	27.62	33.34
	ANOVA P=0.060				
	Tukey's multiple comparisons test		Adjusted P Value		
	NTG vs. TnT-R92Q		0.433		
	NTG vs. TnI-DD		0.583		
	NTG vs. DTG		0.900		
	TnT-R92Q vs. TnI-DD		0.047		
	TnT-R92Q vs. DTG		0.817		
	TnI-DD vs. DTG		0.220		
**Peak Diastolic Velocity (mm/sec)**		**NTG** (n=10)	**TnT-R92Q** (n=8)	**TnI-DD** (n=10)	**DTG** (n=10)
	**Mean**	639.8	534.6	720.3	591.0
	**SE**	63.71	17.68	44.60	57.71
	ANOVA P=0.102				
	Tukey's multiple comparisons test		Adjusted P Value		
	NTG vs. TnT-R92Q		0.512		
	NTG vs. TnI-DD		0.674		
	NTG vs. DTG		0.902		
	TnT-R92Q vs. TnI-DD		0.085		
	TnT-R92Q vs. DTG		0.877		
	TnI-DD vs. DTG		0.284		

<table>
<tr><td rowspan="5">Mean Systolic Velocity (mm/sec)</td><td></td><td>NTG
(n=10)</td><td>TnT-R92Q
(n=8)</td><td>TnI-DD
(n=10)</td><td>DTG
(n=10)</td></tr>
<tr><td>Mean</td><td>104.5</td><td>92.07</td><td>127.9</td><td>72.82</td></tr>
<tr><td>SE</td><td>7.532</td><td>10.95</td><td>8.544</td><td>7.609</td></tr>
<tr><td colspan="5">ANOVA P<0.001</td></tr>
<tr><td colspan="2">Tukey's multiple comparisons test</td><td colspan="3">Individual P Value</td></tr>
<tr><td></td><td colspan="2">NTG vs. TnT-R92Q
NTG vs. TnI-DD
NTG vs. DTG
TnT-R92Q vs. TnI-DD
TnT-R92Q vs. DTG
TnI-DD vs. DTG</td><td colspan="3">0.744
0.243
0.056
0.036
0.409
<0.001</td></tr>
<tr><td rowspan="5">Peak Systolic Velocity (mm/sec)</td><td></td><td>NTG
(n=10)</td><td>TnT-R92Q
(n=8)</td><td>TnI-DD
(n=10)</td><td>DTG
(n=10)</td></tr>
<tr><td>Mean</td><td>161.2</td><td>131.1</td><td>195.2</td><td>111.3</td></tr>
<tr><td>SE</td><td>12.41</td><td>15.21</td><td>13.82</td><td>10.58</td></tr>
<tr><td colspan="5">Kruskal-Wallis test P<0.001</td></tr>
<tr><td colspan="2">Dunn's multiple comparisons test</td><td colspan="3">Adjusted P Value</td></tr>
<tr><td></td><td colspan="2">NTG vs. TnT-R92Q
NTG vs. TnI-DD
NTG vs. DTG
TnT-R92Q vs. TnI-DD
TnT-R92Q vs. DTG
TnI-DD vs. DTG</td><td colspan="3">>0.999
0.954
0.095
0.078
>0.999
<0.001</td></tr>
</table>

NTG, non-transgenic; TnT-R92Q - transgenic mice expressing TnT-R92Q, TnI-DD – transgenic mice expressing TnI-S23,24D, DTG - double transgenic. Data presented as mean ± SEM. n = Sample sizes. AT = acceleration time.

Table 3. Skinned Fiber Bundles Ca^{2+} Force Measurements.

Parameter	Groups			
	**NTG**	**TnT-R92Q**	**TnI-DD**	**DTG**
**n**	8	9	8	8
pCa_{50}	5.73 ± 0.021	6.07 ± 0.033	5.69 ± 0.020	5.98 ± 0.009
	ANOVA P<0.01			
	Tukey's multiple comparisons test		Adjusted P Value	
	NTG vs. TnT-R92Q		<0.001	
	NTG vs. TnI-DD		0.018	
	NTG vs. DTG		<0.001	
	TnT-R92Q vs. TnI-DD		<0.001	
	TnT-R92Q vs. DTG		0.048	
	TnI-DD vs. DTG		<0.001	
Hill Coefficient	4.56± 0.218	3.29 ± 0.273	5.39 ± 0.187	3.79 ± 0.254
	Kruskal-Wallis test P<0.001			
	Dunn's multiple comparisons test		Adjusted P Value	
	NTG vs. TnT-R92Q		0.082	
	NTG vs. TnI-DD		0.750	
	NTG vs. DTG		0.583	
	TnT-R92Q vs. TnI-DD		<0.001	
	TnT-R92Q vs. DTG		>0.999	
	TnI-DD vs. DTG		0.006	
Max Tension (mN/mm^2)	32.00 ± 2.564	23.26 ± 2.286	24.96 ± 2.472	28.00 ± 2.564
	Kruskal-Walllis test P=0.175			
	Dunn's multiple comparisons test		Adjusted P Value	
	NTG vs. TnT-R92Q		0.186	
	NTG vs. TnI-DD		0.957	
	NTG vs. DTG		>0.999	
	TnT-R92Q vs. TnI-DD		>0.999	
	TnT-R92Q vs. DTG		>0.999	
	TnI-DD vs. DTG		>0.999	

NTG, non-transgenic; TnT-R92Q - transgenic mice expressing TnT-R92Q, TnI-DD – transgenic mice expressing TnI-S23,24D, DTG - double transgenic.

Data are presented as mean ± SE. Data were compared using a 1-way ANOVA test, followed by the Tukey test (pCa_{50} data) or Kruskal-Wallis test, followed by Dunn’s test (Hill coefficient and Max Tension data).

Supplemental Table 4. Morphological, Systolic, and Diastolic Parameters Evaluated by Echocardiography at 16 weeks of Age.

Parameter		NTG	TnT-R92Q	TnI-DD	DTG
		**NTG** (n=7)	**TnT-R92Q** (n=6)	**TnI-DD** (n=7)	**DTG** (n=6)
**LA (mm)**	**Mean**	1.65	3.10	1.97	2.14
	**SE**	0.042	0.143	0.098	0.147
	ANOVA P<0.001				
	Tukey's multiple comparisons test		Adjusted P value		
	NTG vs. TnT-R92Q		<0.001		
	NTG vs. TnI-DD		0.186		
	NTG vs. DTG		0.023		
	TnT-R92Q vs. TnI-DD		<0.001		
	TnT-R92Q vs. DTG		<0.001		
	TnI-DD vs. DTG		0.682		
		**NTG** (n=7)	**TnT-R92Q** (n=6)	**TnI-DD** (n=6)	**DTG** (n=6)
**LV mass (mg)**	**Mean**	57.72	86.49	90.42	77.44
	**SE**	1.832	9.212	3.700	7.338
	ANOVA P=0.003				
	Tukey's multiple comparisons test		Adjusted P value		
	NTG vs. TnT-R92Q		0.012		
	NTG vs. TnI-DD		0.004		
	NTG vs. DTG		0.115		
	TnT-R92Q vs. TnI-DD		0.968		
	TnT-R92Q vs. DTG		0.725		
	TnI-DD vs. DTG		0.455		
		**NTG** (n=7)	**TnT-R92Q** (n=6)	**TnI-DD** (n=7)	**DTG** (n=6)
**LVIDd (mm)**	**Mean**	3.93	4.11	3.66	3.61
	**SE**	0.112	0.148	0.203	0.146
	ANOVA P=0.124				
	Tukey's multiple comparisons test		Adjusted P value		
	NTG vs. TnT-R92Q		0.849		
	NTG vs. TnI-DD		0.607		
	NTG vs. DTG		0.485		
	TnT-R92Q vs. TnI-DD		0.217		
	TnT-R92Q vs. DTG		0.162		
	TnI-DD vs. DTG		0.994		
		**NTG** (n=7)	**TnT-R92Q** (n=6)	**TnI-DD** (n=6)	**DTG** (n=6)
**RWT**	**Mean**	0.290	0.350	0.369	0.393
	**SE**	0.0207	0.0144	0.0177	0.0500
	Brown-Forsythe ANOVA test P=0.131; Welch's ANOVA test P=0.077				
	Dunnett's T3 multiple comparisons test		Adjusted P value		
	NTG vs. TnT-R92Q		0.184		
	NTG vs. TnI-DD		0.072		
	NTG vs. DTG		0.392		
	TnT-R92Q vs. TnI-DD		0.938		
	TnT-R92Q vs. DTG		0.942		
	TnI-DD vs. DTG		0.997		

**EF (%)**		**NTG** (n=7)	**TnT-R92Q** (n=6)	**TnI-DD** (n=7)	**DTG** (n=6)
	**Mean**	68.78	67.36	79.18	78.79
	**SE**	1.934	2.112	2.752	1.925
	ANOVA P<0.001				
	Tukey's multiple comparisons test		Adjusted P value		
	NTG vs. TnT-R92Q		0.970		
	NTG vs. TnI-DD		0.013		
	NTG vs. DTG		0.023		
	TnT-R92Q vs. TnI-DD		0.006		
	TnT-R92Q vs. DTG		0.011		
	TnI-DD vs. DTG		>0.999		

**FS (%)**		**NTG** (n=7)	**TnT-R92Q** (n=6)	**TnI-DD** (n=7)	**DTG** (n=6)
	**Mean**	38.03	37.22	47.84	46.83
	**SE**	1.516	1.667	3.032	1.883
	ANOVA P=0.002				
	Tukey's multiple comparisons test		Adjusted P value		
	NTG vs. TnT-R92Q		0.994		
	NTG vs. TnI-DD		0.015		
	NTG vs. DTG		0.042		
	TnT-R92Q vs. TnI-DD		0.011		
	TnT-R92Q vs. DTG		0.030		
	TnI-DD vs. DTG		0.988		

**CO (μl/min)**		**NTG** (n=7)	**TnT-R92Q** (n=6)	**TnI-DD** (n=7)	**DTG** (n=6)
	**Mean**	22.87	23.90	19.87	17.03
	**SE**	1.614	1.363	2.537	2.121
	ANOVA P=0.107				
	Tukey's multiple comparisons test		Adjusted P value		
	NTG vs. TnT-R92Q		0.983		
	NTG vs. TnI-DD		0.693		
	NTG vs. DTG		0.198		
	TnT-R92Q vs. TnI-DD		0.501		
	TnT-R92Q vs. DTG		0.121		
	TnI-DD vs. DTG		0.750		

**HR (bmp)**		**NTG** (n=7)	**TnT-R92Q** (n=6)	**TnI-DD** (n=7)	**DTG** (n=6)
	**Mean**	479.7	478.0	434.0	393.9
	**SE**	19.47	22.43	17.63	33.35
	ANOVA P=0.054				
	Tukey's multiple comparisons test		Adjusted P value		
	NTG vs. TnT-R92Q		>0.999		
	NTG vs. TnI-DD		0.489		
	NTG vs. DTG		0.072		
	TnT-R92Q vs. TnI-DD		0.553		
	TnT-R92Q vs. DTG		0.096		
	TnI-DD vs. DTG		0.624		

SV (μl)		NTG (n=7)	TnT-R92Q (n=6)	TnI-DD (n=7)	DTG (n=6)
	**Mean**	46.71	50.61	45.55	43.66
	**SE**	2.991	3.762	5.063	3.842
	ANOVA P=0.691				
	Tukey's multiple comparisons test		Adjusted P value		
	NTG vs. TnT-R92Q		0.903		
	NTG vs. TnI-DD		0.997		
	NTG vs. DTG		0.950		
	TnT-R92Q vs. TnI-DD		0.813		
	TnT-R92Q vs. DTG		0.650		
	TnI-DD vs. DTG		0.987		

IVRT (msec)		NTG (n=7)	TnT-R92Q (n=6)	TnI-DD (n=6)	DTG (n=6)
	**Mean**	12.21	12.01	12.75	14.08
	**SE**	0.6033	0.7081	1.003	1.048
	ANOVA P=0.336				
	Tukey's multiple comparisons test		Adjusted P value		
	NTG vs. TnT-R92Q		0.998		
	NTG vs. TnI-DD		0.966		
	NTG vs. DTG		0.403		
	TnT-R92Q vs. TnI-DD		0.927		
	TnT-R92Q vs. DTG		0.349		
	TnI-DD vs. DTG		0.701		

E wave (mm/sec)		NTG (n=7)	TnT-R92Q (n=6)	TnI-DD (n=6)	DTG (n=6)
	**Mean**	680.8	648.5	827.1	863.6
	**SE**	23.86	24.06	43.48	85.60
	ANOVA P=0.013				
	Tukey's multiple comparisons test		Adjusted P value		
	NTG vs. TnT-R92Q		0.964		
	NTG vs. TnI-DD		0.174		
	NTG vs. DTG		0.063		
	TnT-R92Q vs. TnI-DD		0.086		
	TnT-R92Q vs. DTG		0.030		
	TnI-DD vs. DTG		0.955		

A wave (mm/sec)		NTG (n=7)	TnT-R92Q (n=6)	TnI-DD (n=6)	DTG (n=6)
	**Mean**	500.2	132.2	363.7	328.1
	**SE**	31.74	19.48	40.18	62.48
	ANOVA P<0.001				
	Tukey's multiple comparisons test		Adjusted P value		
	NTG vs. TnT-R92Q		<0.001		
	NTG vs. TnI-DD		0.108		
	NTG vs. DTG		0.030		
	TnT-R92Q vs. TnI-DD		0.004		
	TnT-R92Q vs. DTG		0.016		
	TnI-DD vs. DTG		0.930		

E/A Ratio

	NTG (n=7)	TnT-R92Q (n=6)	TnI-DD (n=6)	DTG (n=6)
**Mean**	1.383	5.523	2.459	2.979
**SE**	0.0698	0.884	0.3667	0.4777

Brown-Forsythe ANOVA test P=0.002; Welch's ANOVA test P=0.002

Dunnett's T3 multiple comparisons test	Adjusted P value
NTG vs. TnT-R92Q	0.025
NTG vs. TnI-DD	0.146
NTG vs. DTG	0.093
TnT-R92Q vs. TnI-DD	0.073
TnT-R92Q vs. DTG	0.165
TnI-DD vs. DTG	0.935

e' (mm/sec)

	NTG (n=7)	TnT-R92Q (n=6)	TnI-DD (n=6)	DTG (n=6)
**Mean**	22.64	11.44	30.22	26.89
**SE**	1.981	1.100	2.769	2.224

ANOVA P<0.001

Tukey's multiple comparisons test	Adjusted P value
NTG vs. TnT-R92Q	0.005
NTG vs. TnI-DD	0.074
NTG vs. DTG	0.481
TnT-R92Q vs. TnI-DD	<0.001
TnT-R92Q vs. DTG	<0.001
TnI-DD vs. DTG	0.695

E/e' Ratio

	NTG (n=7)	TnT-R92Q (n=7)	TnI-DD (n=6)	DTG (n=6)
**Mean**	30.89	59.43	28.17	33.27
**SE**	1.574	5.967	2.225	4.050

ANOVA P<0.001

Tukey's multiple comparisons test	Adjusted P value
NTG vs. TnT-R92Q	<0.001
NTG vs. TnI-DD	0.952
NTG vs. DTG	0.967
TnT-R92Q vs. TnI-DD	<0.001
TnT-R92Q vs. DTG	<0.001
TnI-DD vs. DTG	0.778

NTG, non-transgenic; TnT-R92Q - transgenic mice expressing TnT-R92Q, TnI-DD – transgenic mice expressing TnI-S23,24D, DTG - double transgenic. Data presented as mean ± SEM. n = Sample sizes. LA = left atrium, LV mass = left ventricle mass, LVIDd = left ventricular internal diameter at diastole, RWT = relative wall thickness, EF = ejection fraction, FS = fractional shortening, CO = cardiac output, HR = heart rate, SV = stroke volume, IVRT = isovolumic relaxation time, E wave = peak velocity of early diastolic transmitral flow, A wave = peak velocity of late diastolic transmitral flow, e' – peak velocity of early diastolic mitral annular motion.

Supplemental Table 5. Antibodies for Western blot and immunohistochemical staining.

Target Antibodies	Cat. number	Supplier	Dilution
WB			
Rb YAP (WB)	14074S	Cell Signaling Technology	1:1000; 5% NFDM + TBST
Rb Phospho-YAP Ser127	4911	Cell Signaling Technology	1::1000; 2% BSA + TBST
Ms Calsequestrin2	Ag13246	Proteintech	1:10000; 5% NFDM + TBST
Ms GATA4	Sc-25310	Santa Cruz	1:100; 5% NFDM + TBST
Rb phospho-GATA4 Ser105	Ab5245	Abcam	1:2000;5% NFDM + TBST
Rb ERK1/ERK2	9102	Cell Signaling Technology	1:1000; 2% BSA + TBST
Rb Phospho-ERK1/ERK2	76299	Abcam	1:2000; 2% BSA + TBST
Ms PLN	A010-14	Badrilla	1:5000; 5% NFDM + TBST
Rb Phospho-PLN Ser16	07-052	EMD Millipore	1:1000; 5% NFDM + TBST
Rb Phospho-PLN Thr17	A010-13	Badrilla	1:2500; 5% NFDM + TBST
Rb CAMKII	A010-56AP	Badrilla	1:2000; 5% NFDM + TBST
Rb Phospho-CAMKII	PA5-37833	Invitrogen	1:1000; 2% BSA + TBST
Rb SERCA2a	A010-23	Badrilla	1:20000; 5% NFDM + TBST
Ms GAPDH (HRP conjugate)	51332	Cell Signaling Technology	1:1000; 2% BSA + TBST

Rb GAPDH	2118	Cell Signaling Technology	1:1000; 2% BSA + TBST
Ms GAPDH	47724	Santa Cruz	1:200; 2% BSA + TBST
Rb alpha/beta Tubulin	2148	Cell Signaling Technology	1:2000; 2% BSA + TBST
Hs-anti-mouse-HRP 2°antibody	7076S	Cell Signaling Technology	1:20,000; 5% NFDM + TBST
Gt-anti-rabbit-HRP 2°antibody	7074S	Cell Signaling Technology	1:20,000; 5% NFDM + TBST
Ms Troponin I	10R-T123K	Fitzgerald Industries International	1:5000; 2% BSA + TBST
Ms Troponin T	564766	BD Biosciences	1:1000; 2% BSA + TBST
Rb MyBP-C	custom	Gift from Rick Moss	1:10000; 5% NFDM + TBST
Ms RLC	ALX-BC-1150-S-L001	Enzo Life Sciences	1:1000; 5% NFDM + TBST
Rt CD31 (IHC)	DIA310	Dianova	1:10; 1%BSA + TBST
Ms α-SMA (IHC)	AB7817	Abcam	1:100; 1%BSA + TBST
IHC			
Rt CD31	DIA310	Dianova	1:10; 1%BSA + TBST
Rb YAP	14074S	Cell signaling Technology	1:100; 1%BSA + TBST
Ms α-SMA	AB7817	Abcam	1:100; 1%BSA + TBST

Gt-anti-rat Alexa Fluor633	A21094	ThermoFisher Scientific	1:1000; 1%BSA + TBST
Gt-anti-rabbit Alexa Fluor568	A11011	ThermoFisher Scientific	1:1000; 1%BSA + TBST
Chicken anti-mouse Alexa Fluor 488	A21206	ThermoFisher Scientific	1:1000; 1%BSA + TBST

Abbreviations used: Ms, mouse antibody; Rb, rabbit antibody; Hs, horse antibody; Gt, goat antibody: NFDM, non-fat dry milk; TBST, Tris-buffered saline with 0.1% (v/v) Tween-20; HRP, horseradish peroxidase; BSA, bovine serum albumin.

References

Alves, M.L., Dias, F.A.L., Gaffin, R.D., Simon, J.N., Montminy, E.M., Biesiadecki, B.J., et al. (2014). Desensitization of myofilaments to Ca2+ as a therapeutic target for hypertrophic cardiomyopathy with mutations in thin filament proteins. *Circ Cardiovasc Genet* 7(2)**,** 132-143. doi: 10.1161/CIRCGENETICS.113.000324.

Batra, A., Warren, C.M., Ke, Y., McCann, M., Halas, M., Capote, A.E., et al. (2021). Deletion of P21-activated kinase-1 induces age-dependent increased visceral adiposity and cardiac dysfunction in female mice. *Mol Cell Biochem* 476(3)**,** 1337-1349. doi: 10.1007/s11010-020-03993-3.

Capote, A.E., Batra, A., Warren, C.M., Chowdhury, S.A.K., Wolska, B.M., Solaro, R.J., et al. (2021). B-arrestin-2 Signaling Is Important to Preserve Cardiac Function During Aging. *Front Physiol* 12**,** 696852. doi: 10.3389/fphys.2021.696852.

Chang, W.T., Fisch, S., Chen, M., Qiu, Y., Cheng, S., and Liao, R. (2015). Ultrasound based assessment of coronary artery flow and coronary flow reserve using the pressure overload model in mice. *J Vis Exp* (98)**,** e52598. doi: 10.3791/52598.

Chowdhury, S.A.K., Warren, C.M., Simon, J.N., Ryba, D.M., Batra, A., Varga, P., et al. (2020). Modifications of Sarcoplasmic Reticulum Function Prevent Progression of Sarcomere-Linked Hypertrophic Cardiomyopathy Despite a Persistent Increase in Myofilament Calcium Response. *Front Physiol* 11**,** 107. doi: 10.3389/fphys.2020.00107.

Fritz, J.D., Swartz, D.R., and Greaser, M.L. (1989). Factors affecting polyacrylamide gel electrophoresis and electroblotting of high-molecular-weight myofibrillar proteins. *Anal Biochem* 180(2)**,** 205-210.

Kinoshita, E., Kinoshita-Kikuta, E., Takiyama, K., and Koike, T. (2006). Phosphate-binding tag, a new tool to visualize phosphorylated proteins. *Mol Cell Proteomics* 5(4)**,** 749-757. doi: 10.1074/mcp.T500024-MCP200.

Matsudaira, P. (1987). Sequence from picomole quantities of proteins electroblotted onto polyvinylidene difluoride membranes. *J Biol Chem* 262(21)**,** 10035-10038.

Solaro, R.J., Pang, D.C., and Briggs, F.N. (1971). The purification of cardiac myofibrils with Triton X-100. *Biochim Biophys Acta* 245(1)**,** 259-262. doi: 0005-2728(71)90033-8 [pii].

Warren, C.M., and Greaser, M.L. (2003). Method for cardiac myosin heavy chain separation by sodium dodecyl sulfate gel electrophoresis. *Anal Biochem* 320(1)**,** 149-151. doi: 10.1016/s0003-2697(03)00350-6.
